# Cell‐type‐specific expression analysis of liver transcriptomics with clinical parameters to decipher the cause of intrahepatic inflammation in chronic hepatitis B

**DOI:** 10.1002/imt2.221

**Published:** 2024-07-04

**Authors:** Jun Wang, Qian Li, Yuanwang Qiu, Simo Kitanovski, Chen Wang, Chenxia Zhang, Fahong Li, Xiaoguang Li, Zhenfeng Zhang, Lihua Huang, Jiming Zhang, Daniel Hoffmann, Mengji Lu, Hongzhou Lu

**Affiliations:** ^1^ National Clinical Research Center for Infectious Diseases The Third People's Hospital of Shenzhen and The Second Affiliated Hospital of Southern University of Science and Technology Shenzhen China; ^2^ Institute of Virology, University Hospital of Essen University of Duisburg‐Essen Essen Germany; ^3^ Clinical Medical Research Center, The Fifth People's Hospital of Wuxi Jiangnan University Wuxi China; ^4^ Bioinformatics and Computational Biophysics, Faculty of Biology and Center for Medical Biotechnology (ZMB) University of Duisburg‐Essen Essen Germany; ^5^ Shanghai Key Laboratory of Infectious Diseases and Biosafety Emergency Response, Department of Infectious Diseases National Medical Center for Infectious Diseases, Shanghai Institute of Infectious Diseases and Biosecurity, Huashan Hospital Fudan University Shanghai China; ^6^ School of Public Health and Emergency Management Southern University of Science and Technology Shenzhen China

**Keywords:** chronic hepatitis B, immune infiltration, innate immunity, population‐specific expression analysis

## Abstract

Functional cure for chronic hepatitis B (CHB) remains challenging due to the lack of direct intervention methods for hepatic inflammation. Multi‐omics research offers a promising approach to understand hepatic inflammation mechanisms in CHB. A Bayesian linear model linked gene expression with clinical parameters, and population‐specific expression analysis (PSEA) refined bulk gene expression into specific cell types across different clinical phases. These models were integrated into our analysis of key factors like inflammatory cells, immune activation, T cell exhaustion, chemokines, receptors, and interferon‐stimulated genes (ISGs). Validation through multi‐immune staining in liver specimens from CHB patients bolstered our findings. In CHB patients, increased gene expression related to immune cell activation and migration was noted. Marker genes of macrophages, T cells, immune‐negative regulators, chemokines, and ISGs showed a positive correlation with serum alanine aminotransferase (ALT) levels but not hepatitis B virus DNA levels. The PSEA model confirmed T cells as the source of exhausted regulators, while macrophages primarily contributed to chemokine expression. Upregulated ISGs (*ISG20, IFI16, TAP2, GBP1, PSMB9*) in the hepatitis phase were associated with T cell and macrophage infiltration and positively correlated with ALT levels. Conversely, another set of ISGs (*IFI44, ISG15, IFI44L, IFI6, MX1*) mainly expressed by hepatocytes and B cells showed no correlation with ALT levels. Our study presents a multi‐omics analysis integrating bulk transcriptomic, single‐cell sequencing data, and clinical data from CHB patients to decipher the cause of intrahepatic inflammation in CHB. The findings confirm that macrophages secrete chemokines like CCL20, recruiting exhausted T cells into liver tissue; concurrently, hepatocyte innate immunity is suppressed, hindering the antiviral effects of ISGs.

## Highlights

- This study integrates liver bulk transcriptomic data, single‐cell sequencing data, and clinical data to analyze the factors inducing hepatic inflammation in chronic hepatitis B from a multi‐omics perspective by Bayesian regression.
- Macrophages secrete chemokines like CCL20 and CXCL8 to recruit immune‐exhausted T lymphocytes (CTLA4, TIGIT) into liver tissue.
- Innate immunity within hepatocytes is suppressed, impeding interferon‐stimulated genes from initiating antiviral effects.
- Activation of innate immune pathways in infiltrating T cells and macrophages further exacerbates inflammation formation.

## Graphical abstract

This study integrates liver bulk transcriptomic data, single‐cell sequencing data, and clinical data to analyze the factors that induce hepatic inflammation in chronic hepatitis B from a multi‐omics perspective by Bayesian regression. Macrophages secrete chemokines like CCL20 and CXCL8 to recruit immune‐exhausted T lymphocytes (CTLA4, TIGIT) into liver tissue. Innate immunity within hepatocytes is suppressed, impeding interferon‐stimulated genes from initiating antiviral effects. Activation of innate immune pathways in infiltrating T cells and macrophages further exacerbates inflammation formation.

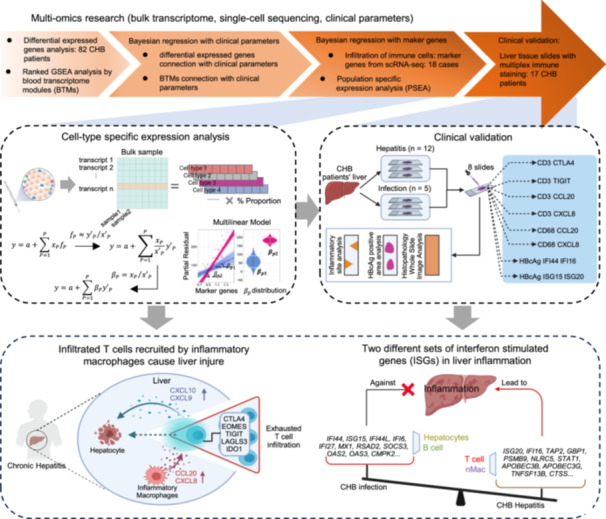

## INTRODUCTION

Worldwide, approximately 260 million people are chronically infected with hepatitis B virus (HBV), resulting in more than 0.7 million deaths per year due to cirrhosis and hepatocellular carcinoma [1]. In persistent HBV infection, host immune responses fail to control the virus, and progressive liver damage, cirrhosis, and cancer may occur [2]. The mechanism of hepatic inflammation formation in chronic hepatitis B (CHB) is not yet fully understood. Although antiviral therapies such as nucleoside analogs combined with interferon are widely used, these treatments cannot eradicate the virus and achieving a functional cure for CHB remains challenging. The difficulty lies in the lack of direct intervention methods for hepatic inflammation.

Multi‐omics research is currently one of the most promising approaches to uncover the mechanisms of hepatic inflammation formation. Initially, Vanwolleghem et al. identified the activation of B lymphocytes and innate immune responses in the peripheral blood transcriptome data of CHB patients during the immune active phase, while T lymphocytes were activated at all stages of CHB [3]. Lebosse et al. further conducted transcriptomic studies in liver tissues of patients with CHB, reporting that in hepatitis B e‐antigen (HBeAg)‐positive patients, the interferon‐stimulated genes (ISGs) of the innate immunity, such as *CXCL10, GBP1, IFITM1, IFNB1, IL10, IL6, ISG15, TLR3, SOCS1*, and *SOCS3*, were suppressed [4]. In 2021, van Buuren et al. reported that in patients with CHB during the immune‐active phase, the interferon pathway was upregulated, immune cells increased, and the importance of immune regulation in achieving a functional cure of HBV was emphasized [5]. In 2022, Montanari et al. also reported the upregulation of ISGs, immune checkpoint genes (*CTLA4, PDCD1, ICOS*), and chemokine genes (*CXCL9, CXCL10*) during the immune‐active phase of CHB [6]. The increased infiltration of immune cells in liver tissue may lead to a significant increase in the proportion of immune cells in biopsy tissue. Consequently, the upregulation of genes is likely due to the increased number of immune cells, resulting in an increase in the average expression level of genes associated with immune cells. Another limitation of using average expression is the inability to fully elucidate the immune pathways involved in inflammation at the cellular level, as well as the specific expression patterns of ISGs in different cell types. Single‐cell sequencing technologies offer a promising way to address the aforementioned limitations. In 2023, Zhang et al. conducted single‐cell sequencing of liver tissue CD45+ immune cells from 23 patients with CHB across various clinical stages. Their findings highlighted the significant role of immune‐exhausted CD8+ T cells in inflammation formation, with FCGR3A+ macrophages facilitating communication with exhausted CD8+ T cells. Nevertheless, the small sample size of patients (only five) in the immune‐active phase presents challenges in constructing correlation statistical models with clinical data [7].

The focus of this study is to integrate liver bulk transcriptomic data with single‐cell sequencing data and clinical data to analyze the inducing factors of hepatic inflammation formation in CHB from a multi‐omics perspective. We developed a Bayesian linear model to establish connections between gene expression and clinical parameters. Additionally, we updated population‐specific expression analysis (PSEA) to deconvolute bulk gene expression into specific cell types during different clinical phases [8]. This was achieved by normalizing the data with cell‐type marker genes identified from single‐cell sequencing data obtained from patients with CHB and healthy liver tissues. Instantly, we integrated these two models into our analysis of the previous issues, such as infiltrating inflammatory cells [9, 10], immune cell activation [11, 12], T cell exhaustion [13, 14, 15], chemokines and receptors [5, 16, 17, 18], innate immunity [19, 20, 21], and ISGs [22, 23, 24, 25]. Furthermore, with the validation of multi‐immune staining in liver specimens from CHB patients, we promptly generated a comprehensive map illustrating the occurrence of hepatic inflammation in CHB.

## RESULTS

### Clustering of CHB patients in four clinical phases

**Figure 1:**
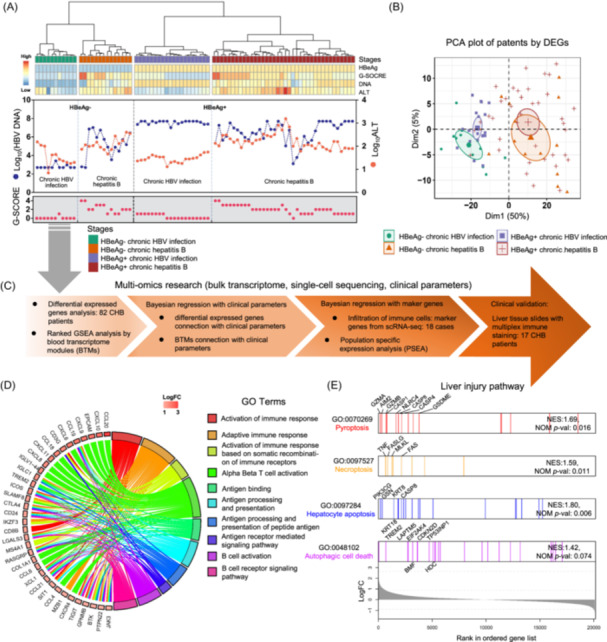
Clustering of chronic hepatitis B (CHB) patients in four clinical phases. (A) Unsupervised clustering for CHB patients into four clinical phases. (B) Principal component analysis of differentially expressed genes. (C) Flowchart of intrahepatic bulk transcriptional analysis to cell‐type‐specific analysis of 82 CHB patients in four clinical phases. (D) Gene set enrichment analysis (GSEA) by ranked gene list according to fold change (hepatitis vs. infection). The chord diagram links the top 10 enriched gene ontology terms and the top 35 upregulated genes. (E) Liver injury pathway enrichment according to the GSEA normalized enrichment score with ranked fold changes. There were 82 CHB patients unsupervised clustered into four clinical stages (natural history) by clinical parameters: immune‐tolerant phase (HBeAg+ infection), immune‐active phase (HBeAg+ hepatitis), inactive carrier phase (HBeAg− infection), and reactivation phase (HBeAg− hepatitis). Differential gene expression analysis and enrichment analysis revealed that upregulated genes during the inflammatory phase were primarily enriched in immune‐related pathways, particularly pathways associated with the recruitment of T lymphocytes. HBeAg, hepatitis B e‐antigen.

The selection of 82 patients has been described previously [26] (Table S1) and included histological examination of liver biopsy samples to exclude patients with fatty liver disease and other liver diseases. The natural progression of CHB unfolds across four distinct phases, characterized by variations in HBeAg status, HBV serum load, and alanine aminotransferase (ALT) levels. During HBeAg‐positive chronic infection, HBV serum load tends to be elevated, and yet, hepatitis is absent. Conversely, in HBeAg‐negative chronic infection, HBV serum load is often underdetected, and hepatitis remains absent. We recruited patients with chronic HBV infection but without immune infiltration and hepatitis as controls, providing a relevant comparison group for investigating the impact of immune cell infiltration on liver hepatitis in the context of HBV infection. We confirmed that patients could be classified by their hepatic gene expression profiles into groups matching classification using clinical parameters. HBeAg status, necro‐inflammatory grading score (*G*‐score) [27], HBV DNA level, and serum ALT level were used to cluster patients into four groups: 19 patients with HBeAg‐positive chronic infection, 38 patients with HBeAg‐positive chronic hepatitis, 11 patients with HBeAg‐negative chronic infection, and 14 patients with HBeAg‐negative chronic hepatitis (Figure 1A). Patients with HBeAg‐positive and ‐negative chronic infection had low *G*‐scores (0−1 points). Patients with HBeAg‐positive chronic infection had high viral DNA loads (10^7^–10^9^ copies/mL) but normal ALT levels. In the majority of patients with HBeAg‐negative chronic infection, DNA was below the detection limit, except in two patients, who had levels of less than 2 × 10^3^ copies/mL. ALT levels were normal in all except three patients with chronic infection. For patients with chronic hepatitis, most (78%) had elevated *G*‐scores of 2−4 points and HBV DNA loads of 10^3^−10^7^ copies/mL, and all presented elevated ALT levels (>40 IU/mL) (Figure 1A).

### Upregulated intrahepatic genes in chronic hepatitis

Principal component analysis (PCA) demonstrated that patients with chronic hepatitis could be distinguished from those with chronic infection based on their hepatic gene expression profiles according to the differentially expressed genes (DEGs) (Figure 1B). However, the HBeAg‐positive or ‐negative status of patients was not distinguished well in the PCA plot. The most upregulated DEGs were found in chronic hepatitis phases, and genes related to immune processes were enriched. The top Gene Ontology terms with top 35 DEGs ranked by fold change (FC) are shown in Figure 1C and all DEGs are shown in Tables S2−S5. Gene set enrichment analysis (GSEA) revealed that DEGs for apoptosis, among the several known pathways of liver injury [28], including pyroptosis, necroptosis, apoptosis, and autophagic cell death, had the highest normalized enrichment scores (Figure 1D). The findings confirm the association between host immune responses and liver injury in chronic hepatitis phases.

In order to annotate DEGs into specific immune functions and cell‐type‐specific processes in the CHB liver, we used enrichment analysis of blood transcriptome modules (BTMs). Each BTM comprises a set of genes with similar expression patterns, annotated with the same biological functions. GSEA, using a pre‐ranked gene list according to FC, was used to enrich all BTMs. Enriched BTMs that were positively correlated with ALT and AST levels included modules for cell cycle, antigen presentation, T cells, monocytes/dendritic cells, NK cells, chemokines, Toll‐like receptor (TLR) signaling, complement, DNA sensing, and IRF2 targets (Figure SB). No clear correlation was observed between most B cell BTMs and ALT or AST levels (Figures S3C and S4).

### Defining marker genes for each intrahepatic cell type

**Figure 2:**
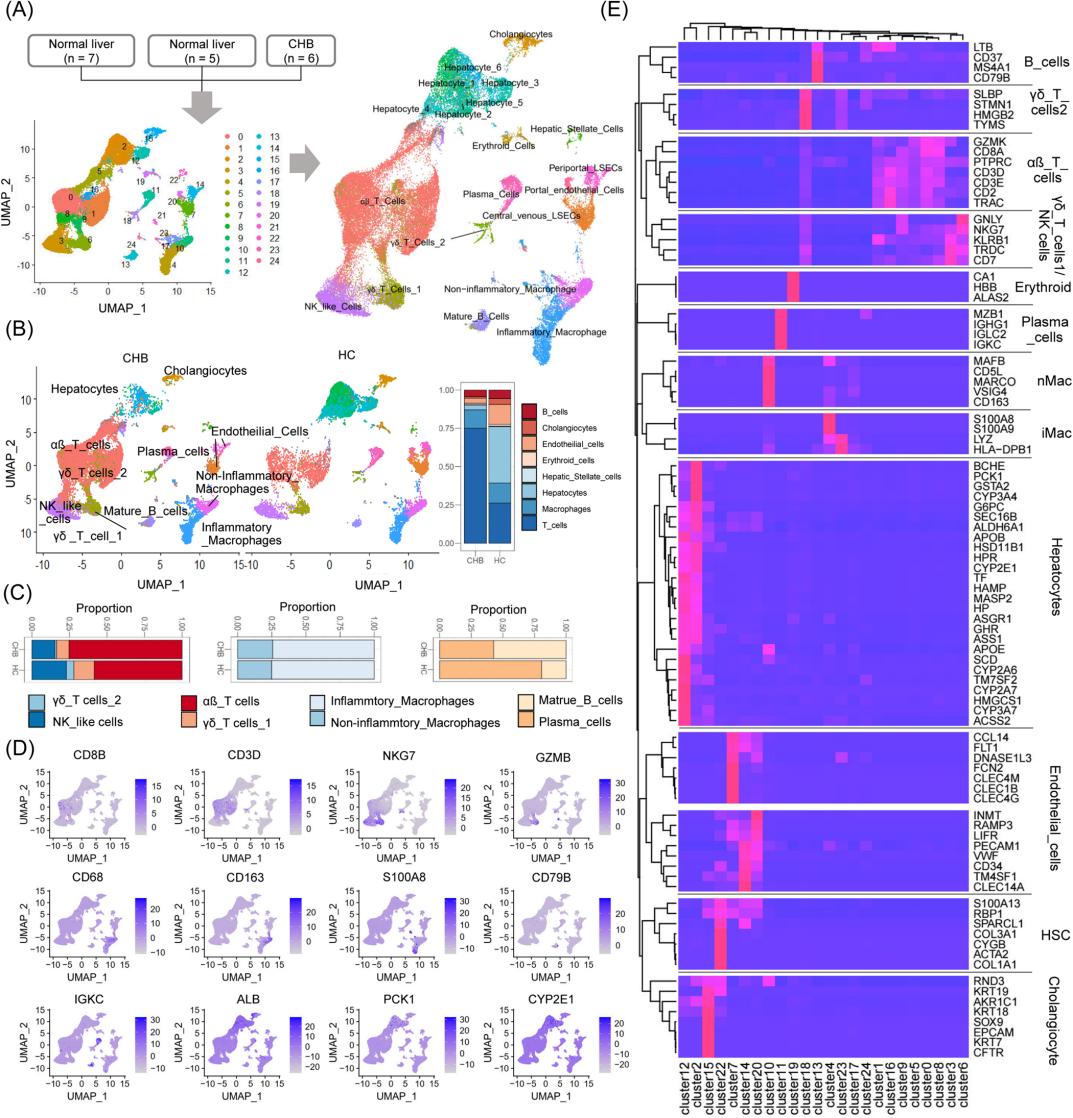
Cell‐type‐specific marker genes in liver based on single‐cell RNA‐sequencing data. (A) Left: Uniform Manifold Approximation and Projection (UMAP) results for visualization and clustering of three merged data sets of 10 × genomic scRNA‐seq from the liver. Right: Twenty cell types were mapped and visualized using UMAP. (B) Left: Clusters representing major cell types in the human liver. Right: Proportions of eight major cell types in CHB and healthy donor livers. (C) Proportions of T cell, macrophage, and B cell subtypes. (D) Projection of known marker genes onto the UMAP plot. (E) Heatmap by hierarchical clustering of 92 intrahepatic marker genes from major cell types in the liver. Thirteen cell types were identified and visualized using the UMAP. These clusters included hepatocytes, cholangiocytes, T cell subtypes (αβ T cells, γδ T cells 1, γδ T cells 2, NK‐like cells), B cells (B cells, plasma cells), macrophages (noninflammatory and inflammatory), and endothelial cells, representing major cell groups in the liver. The proportion of T cells was notably higher in the livers of CHB patients, with αβ T cells being the most abundant T cell subtype, and their numbers were elevated in CHB liver compared to normal donor liver. CHB, chronic hepatitis B.

Three data sets of 10 × genomics single‐cell‐RNA‐sequencing (scRNA‐seq) data from the human liver (GSE115469, GSE186343, GSE124395) were merged and normalized using the ST_transform method, comprising six cases of hepatitis phase CHB liver and 12 health donor livers (Figure 2A,B) [29, 30]. Thirteen cell types were mapped and visualized using the Uniform Manifold Approximation and Projection (UMAP) method. Clusters were designated as hepatocytes, cholangiocytes, T cells (αβ T cells, γδ T cells 1, γδ T cells 2, NK‐like cells), B cells (B cells, plasma cells), macrophages (noninflammatory and inflammatory), and endothelial cells, representing major groups of interest in the liver. The proportion of T cells was higher in the livers of CHB patients (Figure 2C). αβ T cells represented the largest group of T cells in the liver, and their number was increased in the CHB liver as compared with that in the normal donor liver. Known marker genes (*CD8A*, *CD3D*, *NKG7*, *GZMB*, *CD68*, *CD163*, *S100A8*, *CD79B*, *IGKC*, *ALB*, *PCK1*, and *CYP2E1*) were projected onto the UMAP plot (Figure 2D). Hierarchical clustering used to identify intrahepatic cell‐type‐specific marker genes revealed 92 marker genes (Figure 2E).

### Immune cell signatures in CHB and their correlation with liver injury

**Figure 3:**
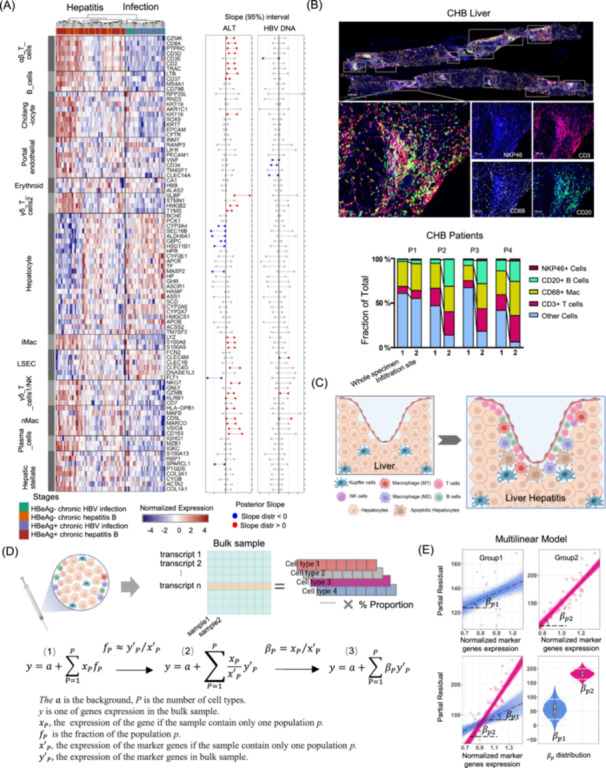
Population‐specific expression analysis to deconvolute bulk gene expression into specific cell types during different CHB phases. (A) Left: expression of top marker genes (rows) for 13 major cell types in the liver for 82 CHB patients (columns in Left). Hepatitis (G3S3, inflammation pathological grade and stage: *G* score = 3, *S* score = 3) and infection phases (G1S1, inflammation pathological grade and stage: *G* score = 1, *S* score = 1) in separate clusters (dendrogram on top). Right: slopes of serum parameters (ALT, HBV DNA) versus marker gene expression (95% credible intervals of marginal posterior); consistently positive and negative slopes are indicated by red and blue bubbles, respectively. (B) Top: Multiplex immune staining of CD3, CD68, CD20, and NKP46 in one representative liver slide from four CHB patients. Bottom: Fraction of CD3+, CD68+, CD20+, and NKP46+ cells in the whole specimen and inflammatory area of representative images from four CHB livers. (C) Schematic of immune cell infiltration from the portal zone into the liver. The image was created using Biorender (https://biorender.com/). (D) Top: Bulk sample transcripts from liver biopsies with percentages of different cell types. Bottom: multi‐linear equation describing relative cell population sizes via expression levels of marker genes (population‐specific expression analysis, PSEA). The image was created using Biorender (https://biorender.com/). (E) Partial residual plots and the fitted linear model in groups, where colored lines for the infection phase (blue) and hepatitis phase (red) represent the corresponding least‐square fits for each group. The violin plot yields the *β* value distribution with a 75% confidence interval for each gene in the two phases, as used to compare the specific expression of genes in the infection (blue dots) and hepatitis phases (red dots). Due to heightened immune cell infiltration during inflammation, gene upregulation stems from cellular activation and increased immune cell numbers, leading to elevated expression of immune cell‐specific genes. Traditional methods cannot discern these influences on gene expression. Updated PSEA to a Bayesian multiple linear regression model utilizing cell‐specific marker gene references. Analysis of the contribution of each cell type to gene expression (coefficient) and evaluation of expression differences of immune‐related genes across different clinical stages for specific cell types (variation in coefficients across clinical stages). ALT, alanine aminotransferase; CHB, chronic hepatitis B; HBV, hepatitis B virus.

Marker genes of T cells (αβ T, γδ T1, γδ T2), NK‐like cells, mature B cells, plasma B cells, and inflammatory and noninflammatory macrophages (iMac and nMac, respectively) were all upregulated in liver samples from the hepatitis phase. The relative expression of hepatocyte marker genes was lower in patients with hepatitis than that in patients with chronic infection (Figure 3A, Left). Plots of marker gene expression versus serum ALT levels and HBV DNA loads showed a positive correlation between ALT levels and the majority of the markers of T cells, iMac, and nMac. In contrast, hepatocyte markers were negatively correlated with ALT levels (Figure 3A, Right). Multiplex immunohistochemical staining of liver biopsies for four chronic hepatitis patients to characterize immuno‐phenotypes and quantify immune cell composition showed that the fraction of lymphocytes was high around the infiltration site at the bile duct, with T and B cell fractions larger at this site than in the whole specimen (Figure 3B, Figure S1). CHB was characterized by intrahepatic infiltration by various kinds of immune cells, which may contribute to liver inflammation (Figure 3C).

Increased expression of immune‐related genes in the liver during the hepatitis phases could be due to either higher immune cell populations in the liver as a result of infiltration or upregulated expression as a result of immune activation. Previous studies have shown that linear models can capture the behavior of a majority of genes [8, 31], and clinical parameters normalized by log10 were approximately normally distributed (Figure S2). RNA expression in a population of cells of a certain type can be described as being linearly dependent on the size of that population. Accurate measurements of the population sizes for different cell types in clinical or autopsy samples are often not possible. However, Kuhn et al. [8] developed a multi‐linear equation that describes relative cell population sizes based on expression levels of marker genes (PSEA; Figure S5). A *β* value with reference to specific marker genes represents normalized gene expression in a certain cell type. Figure 3D shows least‐square fits for the HBeAg‐positive infection phase and the corresponding hepatitis phase, where the slope of the line is the value of *β* derived from the PSEA equation. The violin plot implied a slope distribution with a 75% confidence interval for each gene in the different clinical phases (Figure 3E).

### Chemokines and immune‐exhausted markers highly related with liver injury

**Figure 4:**
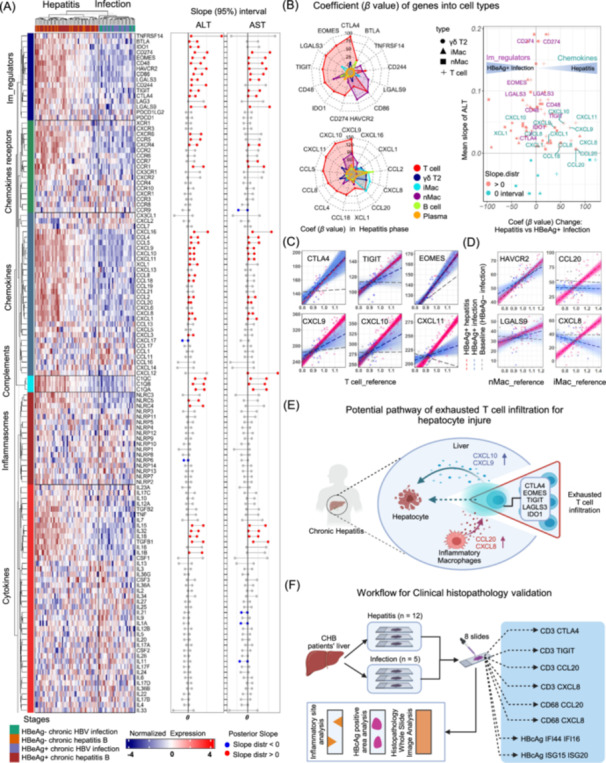
Chemokines and immune‐negative regulators in T cells and macrophages associated with liver injury. (A) Left: Heatmap of gene expression of immune regulation, chemokines, complements, cytokines, and interferons (rows) for all 82 patients (columns) in the four phases of CHB. Right: 95% credible intervals of ALT and AST slopes versus gene expression in Left. Colored bubbles indicate positive (red) or negative slopes (blue) (B) Left: Radar charts of the PSEA coefficient value ($\beta_{P}$) for genes of different reference cell types in chronic hepatitis patients. Right: Changes in gene coefficients of different reference cell types comparing chronic hepatitis to HBeAg‐positive chronic infection. (C, D) Partial residual plot with fitted posterior lines and violin plot of slope distributions from the fitted lines in chronic hepatitis (red) and HBeAg‐positive chronic infection (blue) for gene expression contribution by T cells and iMac cells. Using the established PSEA model, we analyzed upregulated immune checkpoint genes and chemokine genes to discern their predominant expression cell types and expression variations across different clinical stages. (E) Our findings indicated that during the immune‐active phase, immune checkpoint genes (e.g., *CTLA4, EOMES, TIGIT, LGALS3*) were predominantly expressed by infiltrating T lymphocytes, implying that T cell exhaustion drives inflammation. Moreover, chemokine genes strongly correlated with ALT levels, such as *CCL20* and *CXCL8*, were primarily expressed by macrophages, suggesting that macrophages secrete chemokines to recruit immune‐exhausted T lymphocytes into liver tissues. (F) Workflow for clinical histopathology validation: Liver slides from 12 CHB patients in the hepatitis stage and five CHB patients in the infection phase were analyzed. Multiplex immunostaining was performed on eight sequential slides using the parameters outlined in the blue box. These images were created using Biorender (https://biorender.com/). ALT, alanine aminotransferase; CHB, chronic hepatitis B; HBeAg, hepatitis B e‐antigen; PSEA, population‐specific expression analysis.

It is important to understand whether the expression of relevant immune regulatory molecules, cytokines, and chemokines is correlated with ALT levels and/or HBV DNA load. The expression of the majority of genes of negative immune regulation, numerous genes of chemokines, several chemokine receptor genes, genes of the C1Q family, and those of a few specific cytokines was positively correlated with ALT and AST levels but not with serum HBV DNA level (Figure 4A,B, Figure S6A). All these genes were highly expressed in patients with HBV infection. Using PSEA, we deconvoluted the expression of these genes into different cell types and clinical phases. Based on this analysis, we found that (i) expression of coinhibitory genes, including *CTLA4, EOMES, LGALS3, TIGIT, IDO1*, and *CD274* mostly arose from T cells, whereas that of HAVCR2 and LGALS9 arose mainly from nMac, and BTLA was expressed from B cells, (ii) expression of chemokine genes including *CXCL9, CXCL10, CXCL11*, *CCL5, CCL8, CCL4*, and *CCL18* mostly arose from T cells, and (iii) expression of the chemokines *CCL20* and *CXCL8* were mainly from iMac and T cells (Figure 4B, Left). The changes in the coefficients (*β* values) from the HBeAg‐positive infection phase to the hepatitis phase are shown in the plot. *CCL20* from iMac and T cells and *CXCL8* from iMac were the top two upregulated genes in the hepatitis phase (Figure 4B, Right).

Partial residual plots and slope (*β* value) distributions were used to compare the expression of selected genes in specific cell types, including T, iMac, and nMac cells, in the hepatitis and infection phases. The slopes of *CXCL9, CXCL10, CXCL11*, and *TIGIT* were higher in T cells of chronic hepatitis patients than in those of patients with HBeAg+ chronic infection and HBeAg− chronic infection (Figure 4C), indicating that the expression of these genes was increased in the corresponding cell types. The slope distributions of *CTLA4, EOMES*, and *LGALS3* as indicators of T cell exhaustion were narrow and clearly positive in patients with chronic hepatitis and higher than those of patients with HBeAg− chronic infection but typically overlapped in patients with HBeAg+ chronic infection (Figure 4C, Figure S6B‐S6C). Similarly, *HAVCR2* and *LGALS9* expression of nMac in patients with chronic hepatitis overlapped in patients with HBeAg+ chronic infection (Figure 4D). Accordingly, the *β* values for *CCL20* and *CXCL18* in iMac were significantly higher in patients with chronic hepatitis than those in patients with chronic infection (HBeAg− and HBeAg+) (Figure 4D).

Overall, we hypothesized that chemokines, including CXCL8 and CCL20, substantially contribute to the recruitment of exhausted T cells with higher expression of CTLA4, EOMES, TIGIT, LAG3, and IDO1 to the liver by iMacs. The recruited T cells may also release CXCL9 and CXCL10, promoting further T cell infiltration (Figure 4E).

### Pathological validation of markers indicative of T cell exhaustion and chemokines of macrophage activation

**Figure 5:**
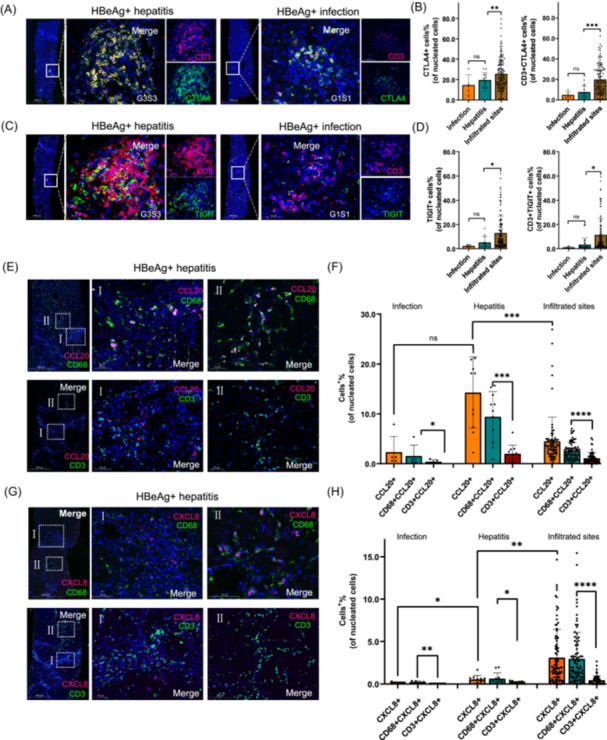
Pathological validation of markers indicative of T cell exhaustion and chemokines of macrophage activation**.** (A) Double immunohistochemical staining of CTLA4 with CD3 in liver specimens of HBeAg‐positive hepatitis patients and HBeAg‐positive infection patients. (B) Column diagram of CTLA4+ and CD3+CTLA4+ cells for CHB hepatitis, CHB infection, and infiltrated site in CHB hepatitis slides. (C) Double immunohistochemical staining of TIGIT with CD3 in liver specimens of HBeAg‐positive hepatitis patients and HBeAg‐positive infection patients. (D) Column diagram of TIGIT+ and CD3+TIGIT+ cells for CHB hepatitis, CHB infection, and infiltrated site in CHB hepatitis slides. (E) Double labeling of CCL20 (red) with CD68 (green) and CD3 (green) in liver specimens from three HBeAg‐positive hepatitis patients. (F) Column diagram of CCL20+, CD3+CCL20+, and CD68+CCL20+ cells for CHB hepatitis, CHB infection, and infiltrated site in CHB hepatitis slides. (G) Double labeling of CXCL8 (red) with CD68 (green) and CD3 (green) in liver specimens from three HBeAg‐positive hepatitis patients. (H) Column diagram of CXCL8+, CD3+CXCL8+, and CD68+CXCL8+ cells for CHB hepatitis, CHB infection, and infiltrated site in CHB hepatitis slides. CTLA4 and TIGIT were colocalized with CD3 at the inflammatory site, suggesting that exhausted CD3+ T cells were widely infiltrated into the liver at these sites during hepatitis. The release of chemokines such as CCL20 and CXCL8 was primarily from CD68+ macrophages and highly expressed in the hepatitis group and inflammatory sites infiltrated by CD3+ T cells, contributing to intrahepatic inflammation. CHB, chronic hepatitis B; HBeAg, hepatitis B e‐antigen.

To validate the results obtained from the multi‐omics analysis, we divided a series of liver slides from 17 patients with CHB into two distinct clinical groups: 12 patients with HBeAg‐positive hepatitis and five patients with HBeAg‐positive infection but without inflammation. Double immunohistochemical staining of CTLA4 and TIGIT with CD3 was performed on these slides. CTLA4 and TIGIT were colocalized with CD3 at the inflammatory site, suggesting that the exhausted CD3+ T cells were widely infiltrated into the liver at infiltrated sites in the hepatitis group (Figure 5A−D).

We also conducted double labeling of CCL20 and CXCL8 with CD68 and CD3, respectively, on liver biopsy samples from HBeAg‐positive hepatitis patients. CCL20 was widely expressed in CD68+ macrophages (Zone I) and partially expressed in CD3+ T cells at the inflammatory site (Zone II) (Figure 5E,F). CXCL8 showed lower expression than CCL20 in the same area of the liver. Similarly, CXCL8 was partially expressed in CD3+ T cells (Zone II) and highly expressed in CD68+ macrophages (Zone I) (Figure 5F, Figures S7 and S8).

### Distinct roles of upregulated ISGs in liver inflammation

**Figure 6:**
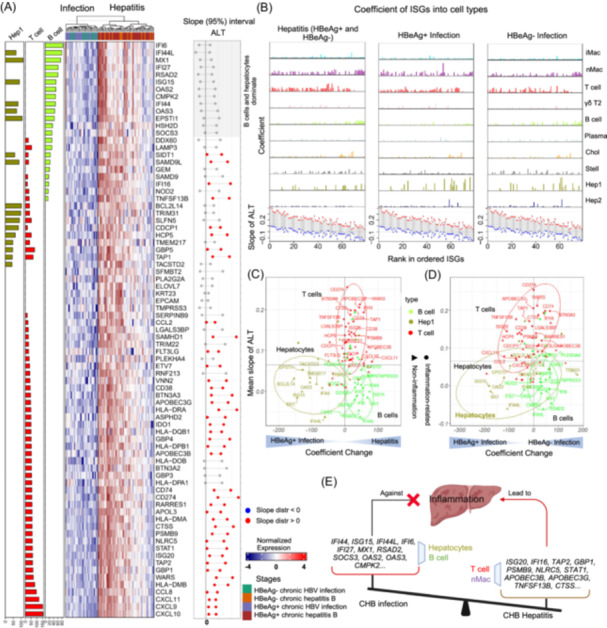
Distinct role of cell‐type‐specific interferon‐stimulated genes in CHB liver inflammation. (A) Expression of 79 ISGs in four phases of CHB (heatmap). Left: ISGs separated according to the *β* value for hepatocyte (Hep1) and T cell gene markers*.* Right: slope probability distribution (95% credible interval) of the linear model of each gene with ALT, with red and blue bubbles indicating positive and negative slopes, respectively. (B) Top: PSEA coefficient (*β*) of 79 ISGs in chronic hepatitis (left), HBeAg‐positive chronic infection (middle), and HBeAg‐negative chronic infection (right) for different cell types. Bottom: 79 ISGs, upregulated in chronic hepatitis patients and ranked by mean slope with ALT; 95% credible intervals for slope distributions. (C) PSEA coefficient (*β*) changes of 45 ISGs in chronic hepatitis groups (HBeAg‐positive and ‐negative) relative to the HBeAg‐positive infection group. (D) PSEA coefficient (*β*) changes of 45 ISGs in the HBeAg‐positive infection group versus the HBeAg‐negative infection group. (E) The hypothesized mechanisms of ISGs primarily expressed by hepatocytes during the HBeAg‐positive infection phase and by B cells during the HBeAg‐negative infection and hepatitis phases, such as IFI44, ISG15, MX1, and OAS3, do not contribute to liver inflammation. Conversely, other ISGs, including ISG20, IFI16, APOBEC3G, CXCL10, and CXCL11, which are predominantly expressed in T cells and nMacs, are implicated in contributing to liver injury. The image was created using Biorender (https://biorender.com/). ALT, alanine aminotransferase; CHB, chronic hepatitis B; HBeAg, hepatitis B e‐antigen; ISGs, interferon‐stimulated genes; PSEA, population‐specific expression analysis.

Whether ISGs are activated during CHB infection remained unclear. To clarify the role of ISGs in chronic HBV infection, we compared the expression of 341 ISGs between CHB patients with chronic hepatitis and those without chronic hepatitis. Of these, 79 ISGs were upregulated in patients with hepatitis, including *STAT1, STAT2, ISG15, ISG20, APOBEC3G, IF16, IFI35, IFI44, OAS2*, and *MX1* (Figure 6A). We used PSEA to analyze the contribution of different hepatic cell types to the expression of these ISGs in all patients with CHB. These ISGs were clustered according to the *β* value and linearized using PSEA for B cells, hepatocytes, and T cell marker references (Figure 6A, Left).

To determine whether ISG expression contributes to viral control and hepatocyte damage, the Bayesian linear model for gene expression with ALT levels revealed a strong positive correlation with ISGs, including *ISG20*, *IFI16*, *TAP2*, *GBP1*, *PSMB9*, *NLRC5*, and *STAT1*, primarily contributed by T lymphocytes and macrophages. Conversely, most ISGs expressed by B lymphocytes and hepatocytes, such as *IFI44*, *ISG15*, *IFI44L*, *IFI6*, *MX1*, and *RSAD2*, showed no correlation with ALT levels (Figure 6A,B). Similar correlations were observed with AST levels. However, there were no significant correlations between HBV DNA and the majority of ISGs (Figure S9A).

We determined the *β* values for these ISGs across different clinical phases. In Figure 6B, ISGs are arranged on the *x*‐axis based on the average slope of their expression in terms of ALT levels (Bottom). Further examination of cellular expression disparities at different clinical stages revealed that during the immune‐active phase (HBeAg+ hepatitis, HBeAg− hepatitis), hepatocytes showed minimal ISG expression (Figure 6B). During this phase, ISGs were predominantly expressed by noninflammatory macrophages and T lymphocytes, with a smaller fraction in B lymphocytes. Additionally, ISGs expressed by T lymphocytes tended to positively correlate with ALT levels, whereas those expressed by B lymphocytes showed no correlation with ALT levels (Figure 6B).

Changes in *β* values for these ISGs per cell type between the HBeAg‐positive infection phase and hepatitis phase are shown in Figure 6C,D. Expression of the top ISGs that correlated with ALT levels, including *APOBEC3G, BTN3A3, CTSS*, and *IFI16*, mostly arose from T and nMac cells. ISGs that were more highly expressed in hepatocytes in the HBeAg‐positive infection phase than in the hepatitis phases, including *IFI44, ISG15, IFI44L, MX1*, and *OAS3*, were not correlated with ALT levels (Figure 6C,D).

Overall, we hypothesized that ISGs primarily expressed by hepatocytes during the HBeAg‐positive infection phase and by B cells during HBeAg‐negative infection and hepatitis phases, such as *IFI44*, *ISG15*, *MX1*, and *OAS3*, showed no correlation with ALT levels. Hence, we inferred that *IFI44* and *ISG15* do not play a role in liver inflammation. Conversely, other ISGs, including *ISG20*, *IFI16*, *APOBEC3G*, *CXCL10*, and *CXCL11*, which were predominantly expressed in T cells and nMacs, and upregulated during the hepatitis phase, showed a positive correlation with serum ALT levels, indicating their involvement in liver injury (Figure 6E, Figure S9B).

### Pathological validation of distinct cell‐type‐specific ISGs in CHB liver

**Figure 7:**
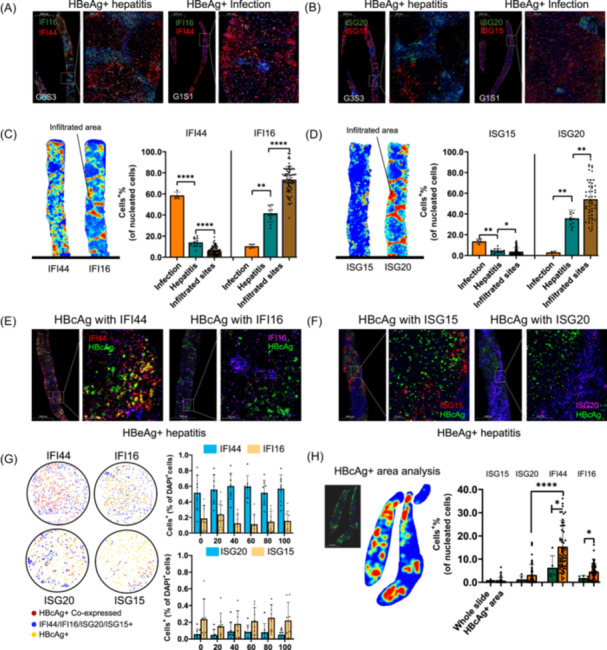
Pathological validation of distinct cell‐type‐specific interferon‐stimulated genes in CHB liver. (A) Double immunohistochemical staining of IFI44 (red) with IFI16 (green) in liver slides from HBeAg+ hepatitis patients and HBeAg+ infection patients. (B) Double immunohistochemical staining of ISG15 (red) with ISG20 (green) in liver slides from HBeAg+ hepatitis patients and HBeAg+ infection patients. (C) Left: Heatmaps illustrating expression levels of IFI44 and IFI16 in hepatitis slides and inflammatory sites based on multiplexed immunofluorescence (mIF). Red indicates higher expression, while blue indicates lower expression. Right: Column diagram of IFI44‐ and IFI16‐positive cells for CHB infection, CHB hepatitis, and infiltrated site in CHB hepatitis slides. (D) Left: Heatmaps illustrating expression levels of ISG15 and ISG20 in hepatitis slides and inflammatory sites based on mIF. Red indicates higher expression, while blue indicates lower expression. Right: Column diagram of ISG15‐ and ISG20‐positive cells for CHB infection, CHB hepatitis, and infiltrated site in CHB hepatitis slides. (E) Double immunohistochemical staining of IFI44 (red) and IFI16 (purple) with HBcAg (green) in liver slides from HBcAg+ CHB patients. (F) Double immunohistochemical staining of ISG15 (red) with ISG20 (purple) with HBcAg (green) in liver slides from HBcAg+ CHB patients. (G) Left: Interaction diagrams showing cellular associations between HBcAg+ cells with IFI44, IFI16, ISG20, and ISG15 for HBcAg‐positive slides. Right: Histogram presenting average distances of cells positive for IFI44, IFI16, ISG20, and ISG15 to HBcAg+ cells within a 100 μm radius. Each bar indicates the percentage of positive cells at different distances. (H) Left: Heatmaps illustrating the distribution of HBcAg+ cells. Heatmaps are used to illustrate the distribution of HBcAg+ cells to identify HBcAg+ areas based on multiplex immunofluorescence (mIF). Red indicates a higher cell count of HBcAg+ cells, while blue indicates a lower cell count of HBcAg+ cells. Right: Histogram presenting the average percentage of double‐positive cells. The histogram presents the average percentage of double‐positive cells for HBcAg+ISG15+, HBcAg+ISG20+, HBcAg+IFI44+, and HBcAg+IFI16+ in the whole slides and specifically in HBcAg+ areas. The *x*‐axis represents the different combinations of double‐positive cells, and the *y*‐axis represents the average percentage of these double‐positive cells. CHB, chronic hepatitis B; HBeAg, hepatitis B e‐antigen.

Double immunohistochemical staining of IFI44 with IFI16 and ISG20 with ISG15 was performed on liver biopsies of 17 CHB patients. IFI44 and ISG15 were more highly expressed in the CHB infection group, while IFI16 and ISG20 were more highly expressed in the CHB hepatitis group. Additionally, IFI16 and ISG20 were primarily expressed at the inflammatory sites of lymphocyte infiltration. In contrast, IFI44 was less expressed at the site of inflammation but widely expressed in hepatocyte‐distributed areas (Figure 7A−D).

Furthermore, we conducted multiple immune staining incorporating ISGs with HBV core antigen (HBcAg); IFI44 was highly colocalized with HBcAg. To elucidate the spatial distribution preferences of HBcAg within CHB liver slides, we used AP‐time software to identify the distribution of HBcAg‐positive cells. the software gauged the spatial proximity (within a distance of <100 µm) between HBcAg‐positive cells and ISGs (IFI44, IFI16, ISG20, and ISG15)‐positive cells within distinct HBcAg‐positive areas (Figure 7G). Furthermore, the heatmap meticulously displayed the areas of HBcAg‐positive cells (Figure 7H). The results indicated that IFI44 was highly expressed in HBcAg‐positive areas, confirming the stimulation of ISGs by the virus across different clinical states.

## DISCUSSION

Understanding the relationship among host immune responses, liver damage, and virus control is central to studies on CHB. Host immune responses are essential for control of viral replication, but in persistent CHB, immune responses fail to control HBV, with the outcome of liver injury [4, 9, 11, 32]. Thus, direct intervention methods for hepatic inflammation are essential to achieve functional cure for CHB.

The increased infiltration of immune cells in liver tissue may lead to a significant increase in the proportion of immune cells in biopsy tissue. The upregulation of genes in previous bulk transcriptomic studies in hepatitis liver tissues is likely due to the increased number of immune cells, resulting in an increase in the average expression level of genes associated with immune cells [3, 4, 5, 6]. Another limitation of using average expression is the inability to fully elucidate the immune pathways involved in inflammation at the cellular level, as well as the specific expression patterns of ISGs in different cell types. Single‐cell sequencing technologies offer a promising way to address the aforementioned limitations. In 2023, Zhang et al. conducted single‐cell sequencing of liver tissue CD45+ immune cells from 23 patients with CHB across various clinical stages. Their findings highlighted the significant role of immune‐exhausted CD8+ T cells in inflammation formation, with FCGR3A+ macrophages facilitating communication with exhausted CD8+ T cells [7]. Nevertheless, the small sample size of patients (only five) in the immune‐active phase presents challenges in constructing correlation statistical models with clinical data. Changes in each cell type in the liver can be quantitatively described using cell‐type‐specific marker genes based on recent single‐cell analysis studies [29, 30], and on this basis, the clinical parameters of liver damage may be correlated with immune cell markers. Thus, we established a Bayesian linear model connecting gene expression with clinical parameters, and a PSEA model deconvolutes bulk gene expression into specific cell types using marker genes from single‐cell sequencing data sets across different clinical phases.

We integrate liver bulk transcriptomic data, single‐cell sequencing data, and clinical data to analyze the factors inducing hepatic inflammation in CHB from a multi‐omics perspective. Data from our analysis in this study are consistent with an earlier proposal [12, 33] that increasing the recruitment of immune cells into the liver causes liver damage without effectively suppressing HBV replication. Persistent HBV infection leads to immune cell exhaustion. CCL20 and CXCL8 from CD68+ macrophages recruit exhausted T cells (CTLA4, EOMES, LGALS3, TIGIT) to the liver, thereby inducing liver injury. Previous studies have shown that CHB patients may have elevated expression of ISGs in liver tissues [34]. Innate immunity within hepatocytes is suppressed, impeding ISGs from initiating antiviral effects. Immune cells are recruited to and activated in the liver during chronic hepatitis phases and may be the dominant cell population expressing specific ISGs. Activation of innate immune pathways in infiltrating immune cells further exacerbates inflammation formation. Thus, intrahepatic cell populations are rather heterogeneous, dynamically change over time, and show uneven spatial distribution in liver tissues. This also explains the expression of different ISGs and their correlation with ALT values.

In the present study, our analysis did not confirm a significant correlation between immunological parameters and HBV DNA levels [26]. This finding is consistent with the hypothesis that not all immune cells recruited to the liver contribute to viral control. In cases where specific T cell responses are not sufficient for HBV control, immune cells, such as macrophages and exhausted T cells, may be recruited. Nonspecific immune cells do not effectively suppress HBV replication but rather cause hepatocyte injury by producing cytotoxic molecules. Therefore, it is understandable that a direct correlation between immune cell markers and HBV DNA levels is not observed in CHB. We conclude that our intrahepatic transcriptome analysis provided evidence for exhausted T cells and macrophage‐driven liver damage but failed to confirm the anti‐HBV effect of hepatic T cell responses in CHB. Activated ISGs in T cells also play an inducible role in hepatitis. However, ISGs contributed by B cells and hepatocytes may serve a protective function against inflammation.

## CONCLUSION

This study proposes three intervention strategies for achieving a functional cure in CHB: Early blockade of liver chemokine secretion, including CCL20 and CXCL8, modulation of intrahepatic T cell status by targeting immune checkpoints or activating specific B cells, and targeted interferon therapy focusing on hepatocytes to induce upregulation of ISGs, thereby exerting antiviral and anti‐inflammatory effects. Alternatively, interferon may induce activation of ISGs in immune cells such as T cells and macrophages, leading to enhanced inflammatory effects.

## METHODS

### Patients' cohort and transcriptome data set

CHB patients (*n* = 82) were recruited and finally included in our study from the Huashan Hospital, Ruijin Hospital, and Public Health Center in Shanghai; the clinical characteristics and following ethical guidelines have been described previously [26] and are presented in Table S1. Unsupervised hierarchical clustering was performed with patients' clinical parameters (serum ALT, HBV DNA, and HBeAg, necro‐inflammatory grading score (0−4) and displayed using R‐package pheatmap) [35]. For clinical validation, we used liver slides from CHB patients; these samples were patient examination residual samples stored in the pathology department. This study was approved by the Research Ethics Committee of The Third People's Hospital of Shenzhen (No. 2024‐051‐04) according to the ethical guidelines of the 1975 Declaration of Helsinki. CHB patients were clustered into two groups: hepatitis (HBeAg+, G ≥ 2) and infection groups (HBeAg+, G < 2); the multiplex immune staining was further conducted to 17 liver slides.

### Differential gene expression analysis

Differential bulk gene expression was assessed using the Limma package [36]. To improve the reliability statistical analysis, we followed the recommendations of the MicroArray Quality Control (MAQC) project and required that significantly DEGs have an adjusted *p* < 0.01 and fold‐changes >2 between two comparison groups [37]. The top two principal components of DEGs with mean and confidence for 82 patients were visualized using R‐package factoextra [38]. With a goal of translating the DEGs into specific immune function‐ and cell‐type‐related annotation, our analysis also used BTMs [39]. Each BTM contains a set of genes annotated with similar biological functions (Supporting Information Materials).

### Defining marker genes for each intrahepatic cell type

Three data sets of 10 × genomics scRNA‐seq data from human liver (GSE115469, GSE186343, GSE124395) were merged and normalized using the ST transform method, comprising six cases of hepatitis phase CHB liver and 12 health donor livers. Thirteen cell types were mapped and visualized using the UMAP method [40]. Candidate marker genes were screened from all DEGs (*p* value < 0.01) of cell clusters from intrahepatic scRNA‐seq data. Then, the method of hierarchical clustering (pheatmap package) was used to explore the part of genes that only expressed in single clusters and the related cell type; these genes were further selected as marker genes for the next analysis.

### Marker‐gene‐based deconvolution of gene expression into cell types with a Bayesian multi‐linear model

Previous studies have shown that linear models may capture the behavior of the majority of genes [31]. Specifically, RNA expression in a population of cells of a certain type can be described as being linearly dependent on the size of that population. As accurate measurements of population sizes of different cell types in human clinical or autopsy samples are often unobtainable, Alexandre et al. provided a multi‐linear equation instead that describes the relative cell population size via the expression levels of marker genes (PSEA) [8]:

$$y=a+\sum_{P=1}^{P}\beta_{P}y_{P}^{\prime }$$

$$\beta_{P}=x_{P}/x_{P}^{\prime }$$

(y is one of the genes expressed in the bulk sample; $y_{P}^{\prime }$ is the expression of the marker gene in population p from the bulk sample; $x_{P}^{\prime }$ is the expression of the marker gene if the sample contains only population p; $x_{P},$ is the specific expression of the gene contributed by the population p; $\beta_{P}$ is the $x_{p}$normalized divided by$\;x_{P}^{\prime }$ in the population p; *a* is the background; and *P* is the number of cell types in the bulk sample).

With $y_{P}^{\prime }$ representing the measured expression of a marker gene of cell population *P* in the bulk data and its value being known, this formulation indeed enables us to construct a multilinear model based on the specific marker gene expression from each cell type to calculate the value of $\beta_{P}$, This process is illustrated in Figure S5A−F.

### Bayesian linear regression to connect gene expression with clinical parameters

To compare the cell‐type‐specific expression of given genes obtained from PSEA in different clinical phases, Bayesian multi‐linear regression was performed (presented in Supporting Information Methods). Bayesian linear regression was also used for exploring the dependency of clinical serum parameters (ALT, AST, and HBV DNA) on normalized expression of enriched BTMs, marker genes of different cell types, and expression of genes of interest, such as immune inhibitors, TLRs, ISGs, chemokines, and receptors, cytokines, and IFNs. We implemented Bayesian linear regression with Stan via R‐package rstanarm function glm with default priors [41].

### Multiplex immune fluorescence staining

Multiplex immune staining was conducted in four liver slides. The optimized concentration of primary antibodies was determined consecutively and combined with corresponding secondary antibodies that were detected using five different fluorescence IHC Kits (Absin Company; abs50029‐20T). The assay was conducted according to the instructions provided. Primary antibodies comprising CD3 (abcam; ab16669), CD20 (abcam; ab78237), CD68 (abcam; ab213363), NKp46 (abcam; ab224703), TIGIT (abcam; ab243903), CTLA4 (CST; #53560S), CXCL8 (CST; #94407S), CCL20 (abcam; ab224188), IFI44 (abcam; ab236657), IFI16 (abcam; ab169788), ISG15 (abcam; ab285367), ISG20 (abcam; ab157477), HBcAg (abcam; ab8639), and DAPI staining solution were bought from Abcam Company (Figures S1, S7, S9).

### Analysis of multiplex immuno‐stained slides

Utilizing AP‐TIME image analysis software (3D Medicines Inc.), images were imported and subjected to rigorous examination. The software facilitated the quantification of cell density, expressed as the count of positively stained cells per square millimeter (cells/mm^2^). Additionally, the software computed the percentage of cells showing positive staining in relation to the total number of DAPI‐stained or nucleated cells, both across the entire liver section and within specific regions of interest. This analysis considered fluorescence across single or multiple channels as appropriate.

To elucidate the spatial distribution preferences of immune cells within the inflammatory site, the software gauged the spatial proximity (within a distance of <100 µL) between cells of interest within distinct cellular lesions. Established cell positivity thresholds for specific markers facilitated precise cell segmentation. Comprehensive data, encompassing each cell's characteristics and spatial coordinates, were extracted for every patient sample. For enhanced visualization and interpretation, the software generated composite images representing the regions of interest based on the collected data. Subsequent data analyses were carried out using GraphPad Prism software (version 8, Inc.). Statistical significance between the two groups was determined using the Mann–Whitney test. Results with an adjusted *p*‐value < 0.05 were deemed statistically significant. Symbols *, **, ***, and ****, respectively, denote *p* < 0.05, *p* < 0.01, *p* < 0.001, and *p* < 0.0001.

## AUTHOR CONTRIBUTIONS

Jun Wang and Qian Li conceived and designed the experiments. Jun Wang and Daniel Hoffmann carried out the data analysis and interpretation. Cheng Wang, Chenxia Zhang, Fahong Li, and Xiaoguang Li collected the data. The manuscript was drafted and revised by Jun Wang, Daniel Hoffmann, Mengji Lu, Qian Li, Simo Kitanovski, and Zhenfeng Zhang. Jiming Zhang, Mengji Lu, Daniel Hoffmann, Lihua Huang, Yuanwang Qiu and Hongzhou Lu contributed reagents, materials, and analysis tools. All authors have read the final manuscript and approved it for publication.

## CONFLICT OF INTEREST STATEMENT

The authors declare no conflict of interest.

## ETHICS STATEMENT

CHB patients were recruited and finally included in our study from the Huashan Hospital, Ruijin Hospital, and the Public Health Center in Shanghai; the clinical characteristics and ethical guidelines were described previously [26]. For clinical validation, we used liver tissue slides from CHB patients; these samples were patient examination residual samples stored in the pathology department and were approved for use by the Research Ethics Committee of The Third People's Hospital of Shenzhen (No. 2024‐051‐04) according to the ethical guidelines of the 1975 Declaration of Helsinki.

## Supporting information


**Figure S1.** Multiplex immune staining of immune cells in the liver of chronic hepatitis B (CHB) patients.
**Figure S2.** Histograms of clinical parameters and gene expressions.
**Figure S3.** Enrichment analysis for blood transcriptome modules (BTMs) linking immune‐related genes to liver injury.
**Figure S4.** Relationship between AST levels with BTM values, comprising marker genes of immune cells and immune processes.
**Figure S5.** Method of population‐specific expression analysis.
**Figure S6.** Correlation between HBV DNA level and upregulated genes’ expression in the phase of Hepatitis.
**Figure S7.** Multiplex immune staining on liver slides and infiltrated area of HBeAg+ hepatitis patients.
**Figure S8.** Multiplex immune staining on liver slides of HBeAg+ infection patients.
**Figure S9.** Correlation between HBV DNA level, AST level, and upregulated genes’ expression in the phase of Hepatitis.

**Table S1.** Clinical information of patients.
**Table S2.** Differentially expressed genes (HBeAg+ Hepatitis vs HBeAg+ infection).
**Table S3.** Differentially expressed genes (HBeAg+ Hepatitis vs HBeAg‐ infection).
**Table S4.** Differentially expressed genes (HBeAg‐ Hepatitis vs HBeAg‐ infection).
**Table S5.** Differentially expressed genes (HBeAg‐ Hepatitis vs HBeAg+ infection).

## Data Availability

The data that support the findings of this study are available from NCBI Gene Expression Omnibus, https://www.ncbi.nlm.nih.gov/geo/, (GEO series accession number: GSE65359, GSE115469, GSE186343, GSE124395) and Supplement data. The scripts used are saved in GitHub https://github.com/JunWang-Lab/iMeta. Supplementary materials (methods, figures, tables, graphical abstract, slides, videos, Chinese translated version, and updated materials) may be found in the online DOI or iMeta Science http://www.imeta.science/.
