# Supplementary material for: Cell‐type‐specific expression analysis of liver transcriptomics with clinical parameters to decipher the cause of intrahepatic inflammation in chronic hepatitis B
Source: Imeta. 2024 Jul 4;3(4):e221. doi: 10.1002/imt2.221 (PMC11316924; doi:10.1002/imt2.221)
Supplement: Supplementary file 1 — Figure S1. Multiplex immune staining of immune cells in the liver of chronic hepatitis B (CHB) patients. Figure S2. Histograms of clinical parameters and gene expressions. Figure S3. Enrichment analysis for blood transcriptome modules (BTMs) linking immune‐related genes to liver injury. Figure S4. Relationship between AST levels with BTM values, comprising marker genes of immune cells and immune processes. Figure S5. Method of population‐specific expression analysis. Figure S6. Correlation between HBV DNA level and upregulated genes’ expression in the phase of Hepatitis. Figure S7. Multiplex immune staining on liver slides and infiltrated area of HBeAg+ hepatitis patients. Figure S8. Multiplex immune staining on liver slides of HBeAg+ infection patients. Figure S9. Correlation between HBV DNA level, AST level, and upregulated genes’ expression in the phase of Hepatitis. [file IMT2-3-e221-s002.docx]

**Supporting information to “Cell-type specific expression analysis of liver transcriptomics with clinical parameters to decipher the cause of intrahepatic inflammation in Chronic Hepatitis B”**

**Running title:** Insights into Hepatic Inflammation in CHB by Multi-Omics

Jun Wang^1,2,3,4#^, Qian Li^1,2#^, Yuanwang Qiu^3#^, Simo Kitanovski^4^, Chen Wang^1^, Chenxia Zhang^3^, Fahong Li^5^, Xiaoguang Li^3^, Zhenfeng Zhang^6^, Lihua Huang^3^, Jiming Zhang^5^*, Daniel Hoffmann^4^*, Mengji Lu^1,2,3^*, Hongzhou Lu^1^*

^1^National Clinical Research Center for Infectious Diseases, The Third People’s Hospital of Shenzhen and The Second Affiliated Hospital of Southern University of Science and Technology, Shenzhen 518112, China.

^2^Institute of Virology, University Hospital of Essen, University of Duisburg-Essen, Essen 45122, Germany.

^3^Clinical Medical Research Center, The Fifth People’s Hospital of Wuxi, Jiangnan University, Wuxi 214007, China.

^4^Bioinformatics and Computational Biophysics, Faculty of Biology and Center for Medical Biotechnology (ZMB), University of Duisburg-Essen, Essen, 45141, Germany.

^5^Department of Infectious Diseases, Shanghai Key Laboratory of Infectious Diseases and Biosafety Emergency Response, Shanghai Institute of Infectious Diseases and Biosecurity, National Medical Center for Infectious Diseases, Huashan Hospital, Fudan University, Shanghai 200040, China.

^6^School of Public Health and Emergency Management, Southern University of Science and Technology, Shenzhen 518055, China.

^#^These authors contributed equally: Jun Wang, Qian Li, Yuanwang Qiu

*Correspondence: [luhongzhou@szsy.sustech.edu.cn](mailto:luhongzhou@szsy.sustech.edu.cn) (Hongzhou Lu); [mengji.lu@uni-due.de](mailto:mengji.lu@uni-due.de) (Mengji Lu); [daniel.hoffmann@uni-due.de](mailto:daniel.hoffmann@uni-due.de) (Daniel Hoffmann); [jmzhang@fudan.edu.cn](mailto:jmzhang@fudan.edu.cn) (Jiming Zhang)

**Supplement Methods**

1. Patients’ cohort and transcriptome dataset………………………………………………….2

2. Bayesian linear regression ….................................................................................................3

3. The introduction of using multiple liner model to explore cell population-specific gene expression …………………………………………………………………………………….4

4. Reference …………………………………………………………………………………...7

**Supplement Figures and Supplement Figure Legends**……………………………………9

**Supplemental Methods**

**1. Patients’ cohort and transcriptome data set**

Liver biopsies and RNA-sequence data initially came from the previous study by Liu, et al. [1] Specifically, eighty-three CHB patients aged at 18–70 years were recruited and selected at the Huashan Hospital, Ruijin Hospital, Renji Hospital, and Public Health Center in Shanghai, China. The study was conformed to the following ethical guidelines (1) written informed consent was obtained from each patient included in the study and (2) the study protocol conforms to the ethical guidelines of the 1975 Declaration of Helsinki as reflected in a priori approval by the institution's human research committee. All patients were excluded according to the following criteria: 1) patients receive antiviral or immunosuppressive therapy < 6 months before sample collection. 2) patients co-infected with other virus, such as hepatitis C virus infection, hepatitis D virus infection, human immunodeficiency virus infection, etc. 3) Patients co-existed with other liver diseases, such as primary biliary cirrhosis, alcoholic liver disease, Wilson disease, or any other coexisting primary liver disease.

RNA was extracted from liver biopsies of these 83 CHB patients. Microarray analysis was performed using the Aﬀymetrix Human Genome U133 Plus 2.0 Array. The microarray data was normalized by robust multichip averaging (RMA), and data deposited in NCBI Gene Expression Omnibus (GEO series accession number GSE65359). The top 2 principal components of all samples were calculated by principal component analysis (PCA) to check the normalized data, revealing one outlier sample (GSM1593160) that was then excluded from further analysis. Next, the normalized gene expression matrix of 82 patients was processed as follows: (1) probe sets without gene symbols annotation were omitted; (2) probes targeting multiple genes were omitted; (3) if a gene was targeted by multiple probes, only the probe with the maximum mean signal was retained.

**2. Bayesian linear regression Posterior slope distribution of clinical parameters (ALT, AST, HBV DNA) contributed by the normalized value of enriched BTMs.**

Firstly, clinical parameters normalized by log_10_, were approximately normally distributed. The normalized value of BTMs covered a much smaller range and were normally distributed without further transformation. To quantify the dependency of log_10_ALT, log_10_ AST and log_10_ (HBV DNA) on normalized values of BTMs, respectively. We performed linear regression in Stan via R-package rstanarm. Models were treated formally as generalized linear models with the identity link function and Gaussian noise.

**Posterior slope distribution of clinical parameters (ALT, AST, HBV DNA) dependent on gene expression, respectively.**

The values of genes expression were normalized by log2 and were approximately normally distributed without further transformation, we displayed distribution of several genes.

The priors for all models were used as following: For the intercepts Gaussian priors with location 0 and scale 10, for the standard deviation an exponential with rate 1, and for the covariance a decov prior with regularization, concentration, shape, and scale all set to 1.

Convergence of Markov Chain Monte Carlo (4 chains, each with 1000 steps warm-up and 1000 steps sampling) was checked by inspection of Gelman-Rubin parameter R, which was close to one for all considered models, typically < 1.01 [2]. Model ability to generalize was tested by approximate leave-one-out cross-validation, where all Pareto k estimates were good (k < 0.5) [3].

**Host immune responses as major contributors to liver damage**

In order to annotate DEGs into specific immune functions and cell-type-specific processes in CHB liver, we used enrichment analysis of blood transcriptome modules (BTMs). Each BTM comprises a set of genes with similar expression patterns, annotated with the same biological functions. GSEA, using a pre-ranked gene list according to FC, was used to enrich all BTMs. The mean value of the normalized gene expression in each module was taken as module expression. A linear model was used to analyze the relationship between clinical parameters and module expression; for example, we used the slopes of fitted models to quantify the strength of the relationship between liver injury (as determined based on ALT and aspartate aminotransferase (AST), another marker of liver injury) and module expression (Figure S3A). Enriched BTMs that were positively correlated with ALT and AST levels included modules for cell cycle, antigen presentation, T cells, monocytes/dendritic cells, NK cells, chemokines, TLR signaling, complement, DNA sensing, and IRF2 targets (Figure S3B). No clear correlation between most B cell BTMs and ALT or AST levels was observed; BTMs with strong correlations with ALT and AST levels also had high normalized enrichment scores (Figure S3C, Figure S4). Importantly, few correlations between the listed BTMs and HBV DNA load were observed, although three BTMs (one for B cells and two for leukocyte migration) showed a negative correlation with HBV DNA levels. Therefore, host immune activation in the liver may contribute to liver injury without necessarily controlling HBV replication.

**3. The introduction of using multiple liner model to explore the expression of specific genes contributed by specific cell-types according to the method of population specific expression analysis (PSEA)** [4, 5]

**Equations:**

When measuring gene expression from a bulk sample composed of multiple cell types, the observed gene expression is contributed by these different cell populations. Specifically, the measured gene expression is the sum of each cell type’s contribution, where each cell type’s contribution is proportional to both the cell population and the specific gene expression within that population.

$$y=a+\sum_{P=1}^{P} {x_{P}f}_{P} (Equation 1)$$

(Here, $a$represents the background expression, $x_{P}$ represents the specific expression of a gene in population P, and $f_{P}$ represents the fraction of population P). However, However, in most cases, the fraction of each cell type ($f_{P}$) is unknown. To address this, we can utilize specific marker genes that are uniquely expressed in one cell type. In this scenario, the measured expression of marker genes is only contributed by that particular cell population, and the marker genes exhibit high expression compared to the background. Consequently, we have:

${{y'}_{P} \approx{x'}_{P}f}_{P} (Equation 2)$

(Here, ${y'}_{P}$ represents the measured expression of a marker gene of cell population $P$ in the bulk data, and ${x'}_{P}$ represents the expression of marker gene if the sample is composed by only this cell population $P$)

By utilizing Equation 2, we can estimate the fraction of each cell type:

$f_{P}{\approx{y'}_{P} / {x'}_{P}}(Equation 3)$

Combining Equation 1 and Equation 3, we obtain:

$$y=a+\sum_{P=1}^{P} x_{P}\frac{{y'}_{P}}{{x'}_{P}} (\mathrm{Equation}4)$$

Finally, when we define $\beta_{P}={x_{P}}/{x_{P'}}$, we arrive at the final equation:

$$y =a+ \sum_{P=1}^{P} \beta_{P}{y'}_{P} (\mathrm{Equation}5)$$

($\beta_{P}$ represents the­ specific­ expression­ of ­the­ gene­ ­in population­ $P$ normalized­ to­ the­ specific­ expression­ of­ the­ marker gene ­in population­ $P$­) (Figure S5A).

With ${y'}_{P}$ representing the measured expression of a marker gene of cell population $P$ in the bulk data, and its value being known, this formulation indeed enables us to construct a multilinear model based on the specific marker gene expression from each cell type to calculate the value of $\beta_{P}$, This process is illustrated in Figure S5A.

**Pareto optimization:**

Pareto frontier optimization in the PSEA method serves to address the challenges posed by multi-objective optimization when selecting marker genes for known cell types. In our method, multiple marker genes may exist for a single cell type, leading to a set of potential marker genes (represented by ${y'}_{P}$). Consequently, the task becomes one of selecting an optimal subset of marker genes from this set, taking into consideration multiple objectives such as maximizing the informativeness of the selected markers while minimizing redundancy and false discoveries. Due to the expression of marker genes from the same cell population should be well correlated in the bulk sample, such as, the transcriptome data from our 82 CHB patients. For the PSEA method, the highly related marker genes should be selected to be the marker reference for one cell type. Partial correlation matrix was firstly performed to screen marker genes with highly correlation (R values > 0.8) based on the method of Spearman. Marker genes in the box of a distinctive cluster were highly correlated with each other in specific cell type (Figure S5B).

Secondly, the candidate marker genes in specific cell type should be filtered as quite higher correlation with other marker genes inside the box and relatively lower correlation with marker genes from other cell types. Thus, we used an average R value from a specific marker gene with other marker genes inside the box (In_R) and the highest R value of this marker gene with marker genes from other cell types (Out_R). It turned to be an issue of multi-objective optimization, one way to find good solutions is Pareto frontier optimization [6]. The Pareto frontier solution was used to define the optimal marker genes with higher In_R and lower Out_R (Figure S5C). The first Pareto frontier was determined by the points which were not dominated by others. In the same way, the second Pareto frontier was determined after removal of the first one. In the end, all the Pareto points were mapped as a boundary representing the set of selected marker genes for marker reference from Pareto optimization. The Pareto frontier was visualized by geom_frontier method from package “KraljicMatrix” [7].

**Marker-genes-reference:**

Cell specific marker reference was calculated in PSEA method as follows: each marker gene was normalized to a mean value of 1 in patients. Then, the normalized expression of all selected marker genes within each cell type were averaged as population-specific reference signals to maintain an equal weight (Figure S5D).

**StepAIC:**

The expression of genes from the bulk sample were usually not contributed by all included cell populations. Thus, the issue of which cell type reference should be included in the multilinear model need to be selected firstly. The PSEA method used a classical stepwise selection method based on Akaike’s AIC criterion [8]. Then the variables of cell marker reference were selected to find which contributed to specific gene expression. The function stepAIC (MASS package) [9] was performed. We fitted the model with lowest AIC for variables selection. Then discard genes whose response variability was poorly explained the statistical model (adjusted R^2^ ≤ 0.3). Extract coefficients with *p* < 0.05 for the final selected variables (Figure S5E). The model linearity was finally checked by partial residual plots [10].

**Partial residual plots:**

Partial residual plots were used to show the relationship between a given marker reference and the specific gene expression contributed by this marker reference represented cell type in the multilinear model. Partial residual plots are formed as:

$Res+\beta_{i}X_{i} versus X_{i}$

(Res = residuals from the full model, $\beta_{i}$ = regression coefficient from the i-th independent variable in the full model, $X_{i}$ = the i-th independent variable, the value of partial residual also called Component Plus Residual (CR)) [11]

The $\beta_{i}$ was the same with $\beta_{P}$ which was the normalized­ gene expression­ ­in specific cell type­ $P$­. Thus, the next step for the comparison of each gene expression­ ­in specific cell type­ between different groups can be transformed to compare $\beta_{i}$ from different groups in the partial residual plot.

**Posterior** $\boldsymbol{\beta}_{\boldsymbol{i}}$ **distribution from different groups in the partial residual plot**:

We performed Bayesian linear model to calculate the posterior β_i distribution from different groups in the partial residual plot, Measurements were checked by visual posterior predictive checks to get a comprehensive diagnostic result instead of residual normality, homogeneity of variance, and other assessments. Models were treated formally as generalized linear models with the identity link function and Gaussian noise. The Bayesian linear regression was implemented in Stan via R-package rstanarm. [12, 13] In the notation of the linear models for component plus residual (CR) as variate and the specific marker reference as covariate in different clinical groups, CR ~ (specific marker reference | hepatitis patients) and CR ~ (specific marker reference | infection patients). Models were treated formally as generalized linear models with the identity link function and Gaussian noise. Measurements were checked by visual posterior predictive checks.

Colored lines of infection phase (Blue line) and hepatitis phase (Red line) are the corresponding least-square fits form each group. The violin plot implied the slope distribution with a 75% confidence interval of each gene in two phases was used to compare cell-type specific expression in infection phase (Blue dots) and hepatitis phase (Red dots) (Figure S5F).

**REFERENCES**

1. Hongyan Liu, Fahong Li, Xiaoyong Zhang, Jie Yu, Jinyu Wang, Jia Jia, Xueping Yu, et al. 2018. “Differentially Expressed Intrahepatic Genes Contribute to Control of Hepatitis B Virus Replication in the Inactive Carrier Phase.” *Journal of Infectious Diseases* 217: 1044-1054. <https://doi.org/10.1093/infdis/jix683>

2. Douglas Bates, Martin Mächler, Ben Bolker, Steve Walker, 2015. 'Fitting Linear Mixed-Effects Models Using lme4. ', Journal of Statistical Software.

3. Aki Vehtari, Andrew Gelman, Jonah Gabry. 2017. 'Practical Bayesian model evaluation using leave-one-out cross-validation and WAIC', Statistics and Computing.

4. Alexandre Kuhn, Doris Thu, Henry J Waldvogel, Richard L M Faull, Ruth Luthi-Carter. 2011. “Population-specific expression analysis (PSEA) reveals molecular changes in diseased brain.” *Nature Methods* 8: 945-947. <https://doi.org/10.1038/nmeth.1710>

5. Alexandre Kuhn, Azad Kumar, Alexandra Beilina, Allissa Dillman, Mark R Cookson, Andrew B Singleton. 2012. “Cell population-specific expression analysis of human cerebellum.” *BMC Genomics* 13: 610. <https://doi.org/10.1186/1471-2164-13-610>

6. Kaisa Miettinen. 1999. “Nonlinear Multi-objective Optimisation. Kluwer Int.” *Series*, <https://joss.theoj.org/papers/10.21105/joss.00170>

7. Bradley C BoehmkeRobert T. MontgomeryJeffrey A. OgdenJason FreelsJason Freels. 2017. 'KraljicMatrix: An R package for implementing the Kraljic Matrix to strategically analyze a firm’s purchasing portfolio', Journal of Open Source Software.

8. Akaike, H. 1974. 'A new look at the statistical model identification', *IEEE Transactions on Automatic Control*, pp. 716 - 723.

9. W.N.Venables, B.D.Ripley. 2002. 'Modern Applied Statistics with S. ', *Statistics and Computing*.

10. Mark J. Dunning, Mike L. Smith, Matthew E. Ritchie, Simon Tavaré. 2007. “beadarray: R classes and methods for Illumina bead-based data.” *Bioinformatics* 23: 2183-2184. <https://doi.org/10.1093/bioinformatics/btm311>

11. Velleman, Paul F., Roy E. Welsch. 1981. “Efficient Computing of Regression Diagnostics.” *JSTOR* 35: 235-242. <https://doi.org/10.2307/2683296>

12. Bob Carpenter, Andrew Gelman, Matthew D. Hoffman, Daniel Lee, Ben Goodrich, Michael Betancourt, Marcus Brubaker, et al. 2017. “Stan: A Probabilistic Programming Language.” *J Stat Softw* 76: <https://doi.org/10.18637/jss.v076.i01>

13. Goodrich B, Gabry J, Ali I & Brilleman S. 2020. 'rstanarm: Bayesian applied regression modeling via Stan'.

**Supplement Figures and Supplement Figures Legends**

**Figure S1**


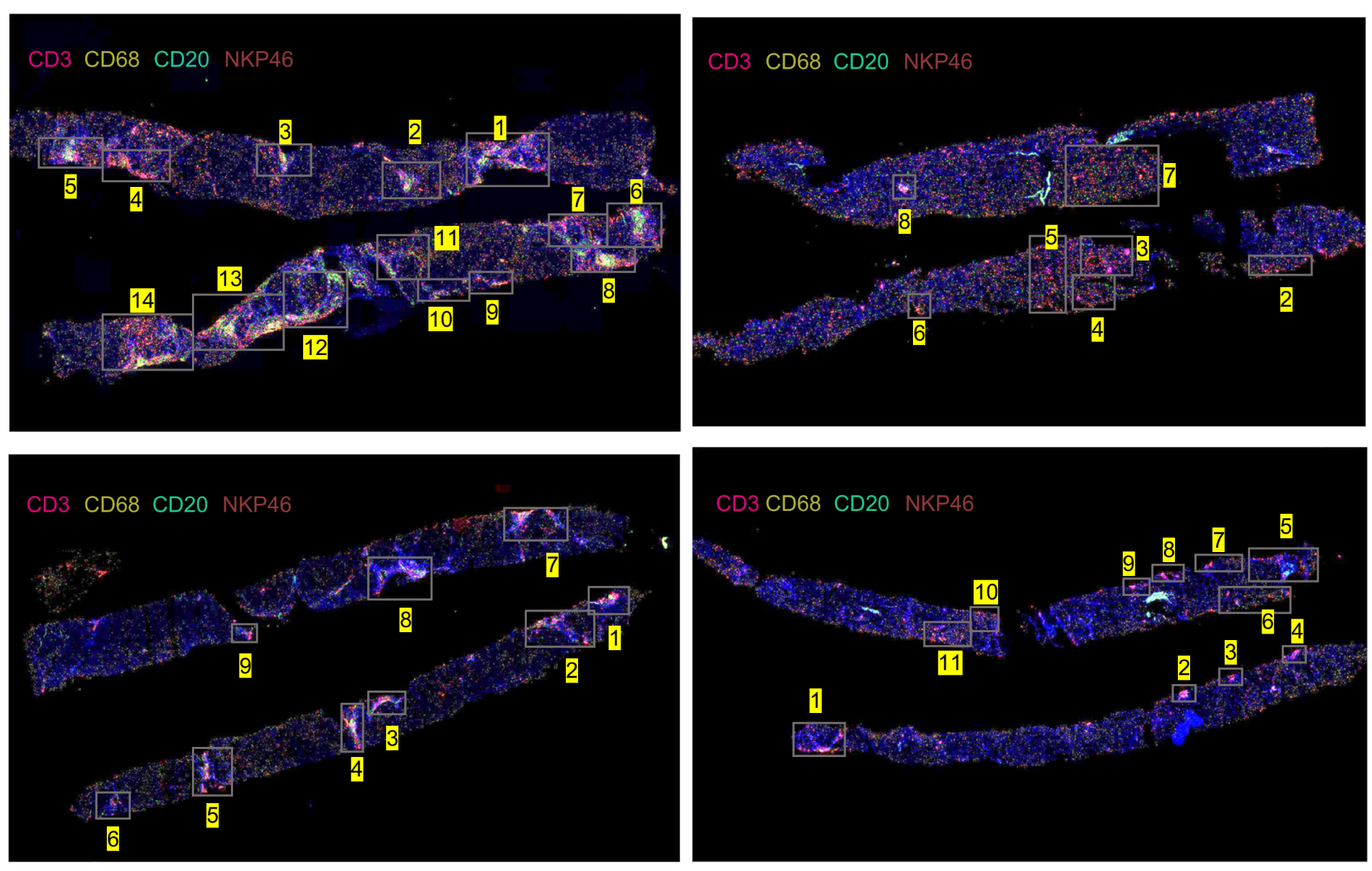


**Figure S1. Multiplex immune staining of immune cells in the liver of chronic hepatitis B (CHB) patients.**

Multiplex staining on slides from liver biopsies of CHB patients. Slides were stained with CD3 (pink), CD20 (green), CD68 (yellow), NKP46 (red) and DAPI (blue) in order and were merged after Ag retrieval. Original magnification × 20. The inflammation site with labeled numbers were counted to numbers of CD3, CD20, CD68, NKP46 and DAPI positive cells.

**Figure S2**


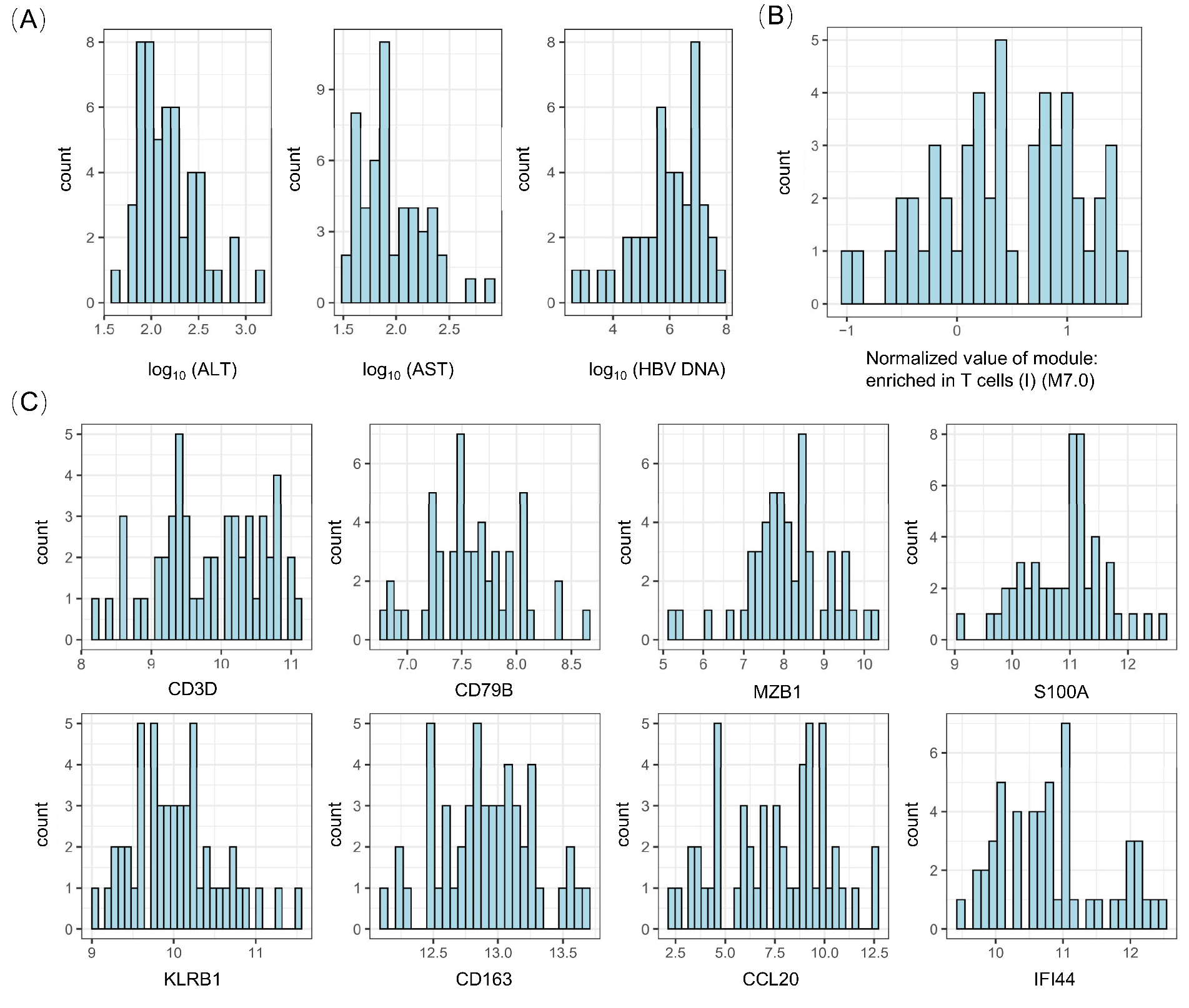


**Figure S2. Histograms of clinical parameters and genes expressions**

(A) Histograms of clinical parameters (log_10_(ALT), log_10_(AST), log_10_(HBV DNA)). (B) Histograms of one normalized value (Module: enriched in T cells, M7.0). (C) Histograms of several genes normalized expression.

**Figure S3**


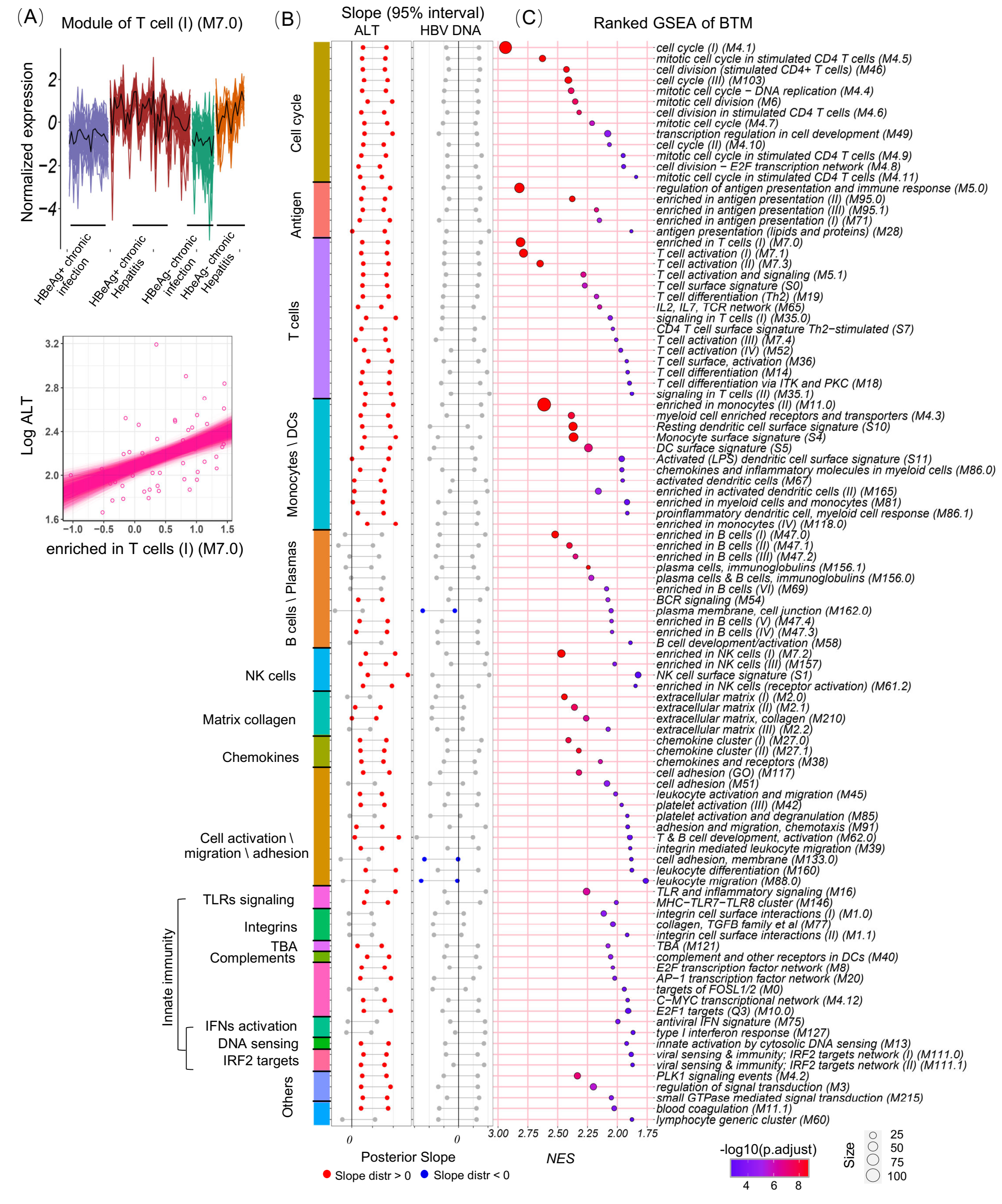


**Figure S3. Enrichment analysis for blood transcriptome modules (BTMs) linking immune-related genes to liver injury**

(A) *Upper panel*: mean value (black line) of T cell module expression (M = 7.1 BTM) in four clinical phases. *Lower panel*: distribution of fitted lines for linear model correlating log ALT with T cell module expression. (B) Slopes (95% credible interval) of linear models correlating ALT (left panel) and HBV DNA (right panel) with module expression. Colors of points at interval bounds indicate consistently negative (blue) or positive (red) slopes. (C) Bubble size represents the number of genes in the module. Bubble colors represent -log_10_ of the adjusted P values.

**Figure S4**


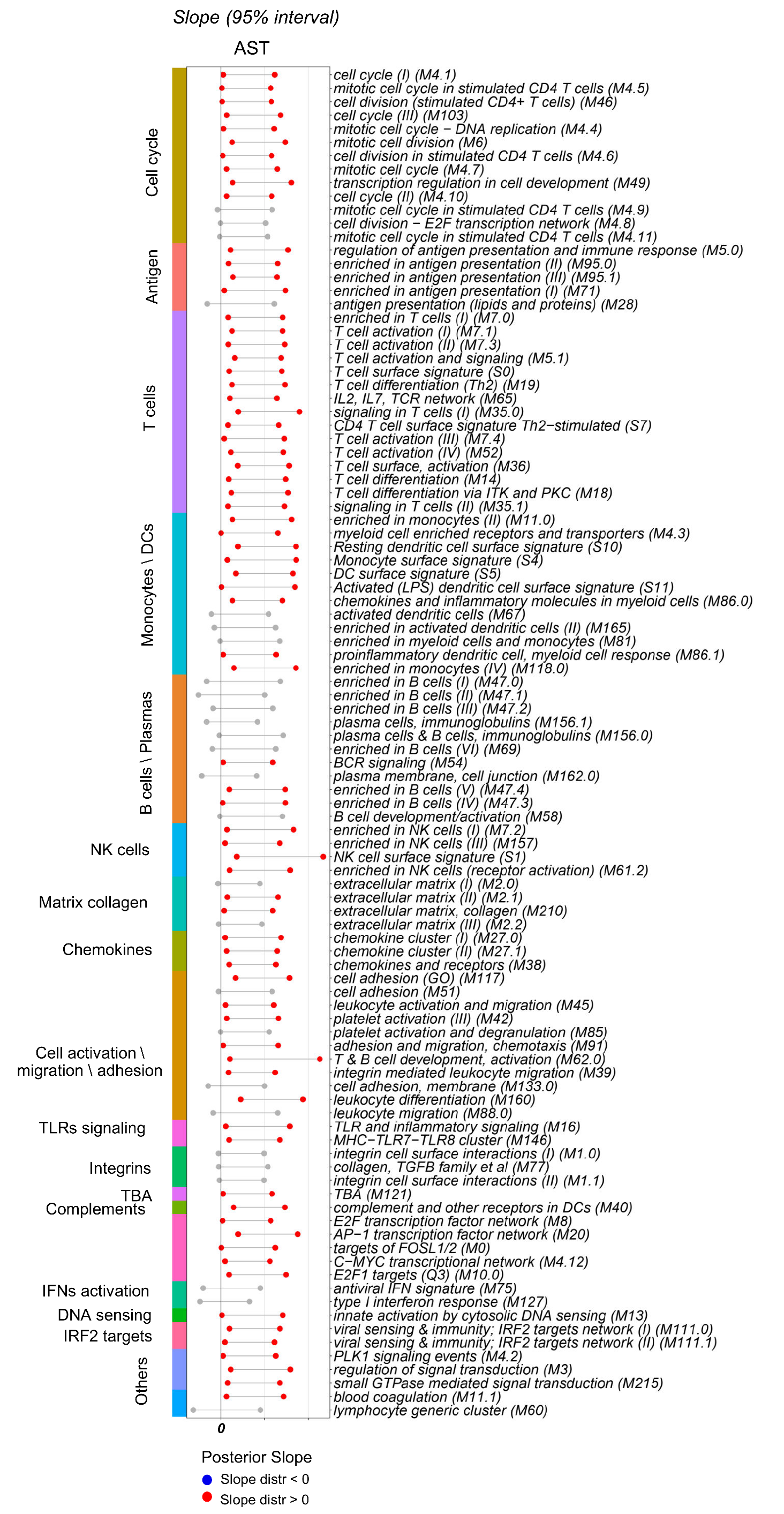


**Figure S4. The relationship between AST levels with BTM values, comprising marker genes of immune cells and immune processes.**

The relationship between AST with enriched BTM values. The slope distribution (95% confidence interval) of the fitted line of each BTM value with AST.

**Figure S5**


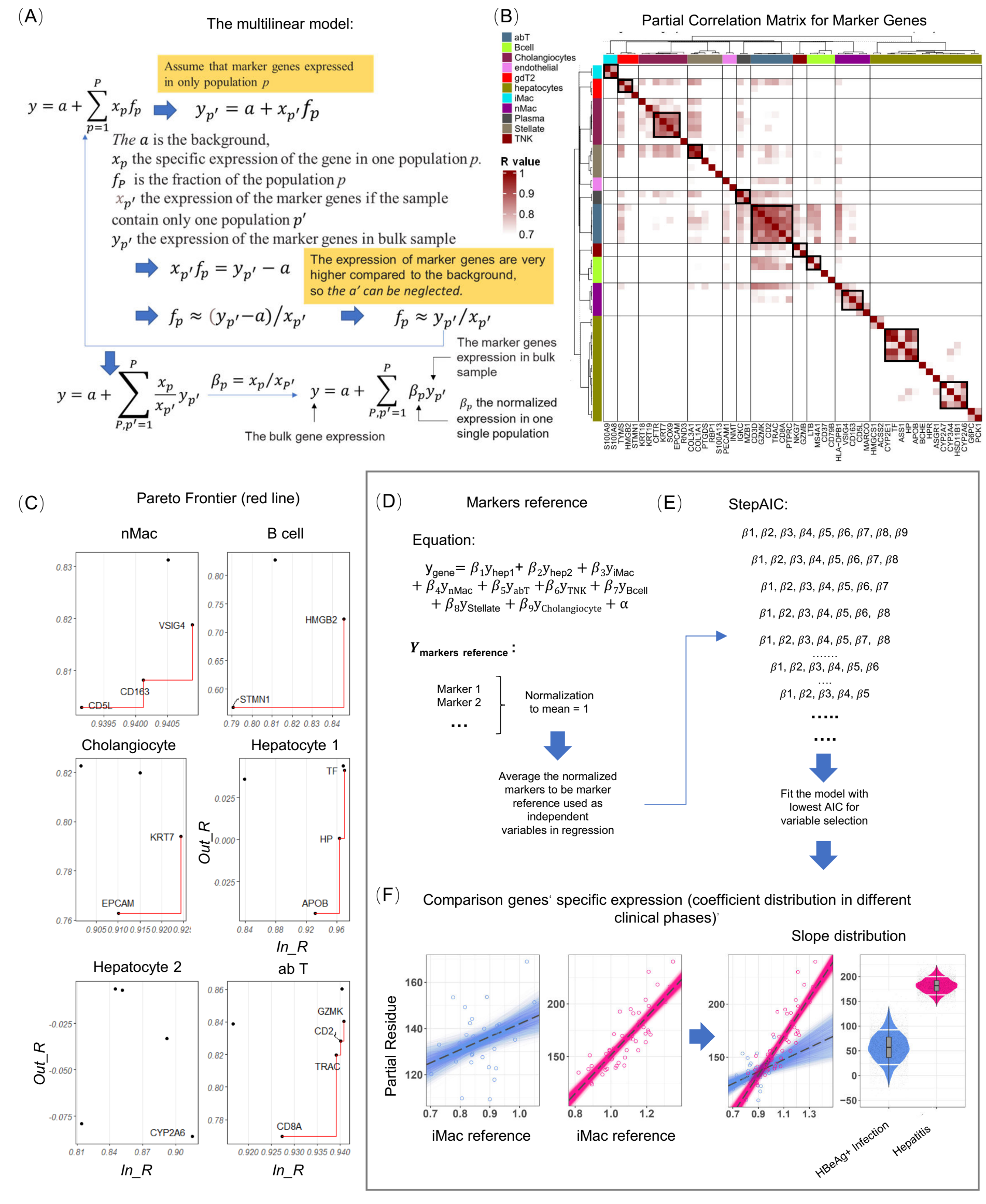


**Figure S5. The method of population specific expression analysis**

(A) The equation of multilinear model of population specific expression analysis. (B) Partial correlation matrix. Genes in the box indicated for a highly correlated genes from specific cell type. (C) The x-axis “*In-R*” for the Pareto Frontier Graph indicates an average R Value of correlations between a marker gene with other marker genes in the box of Partial Correlation Matrix, y-axis “*Out-R*” means the highest R value from correlations between a marker gene in the box with marker genes of other cell types. The mapped red line was known as Pareto frontier, which is the boundary plotted by the current optimal points. (D) The workflow of marker reference signals, which was further used ­as­ independent variables­ in multilinear regression. (E) The workflow of stepAIC for feature selection. (F) Partial residual plots and the fitted linear model in groups, colored lines of infection phase (Blue line) and hepatitis phase (Red line) are the corresponding least-square fits form each group. The violin plot implied the slope distribution with a 75% confidence interval of each gene in two phases was used to compare genes’ specific expression in infection phase (Blue dots) and hepatitis phase (Red dots).

**Figure S6**


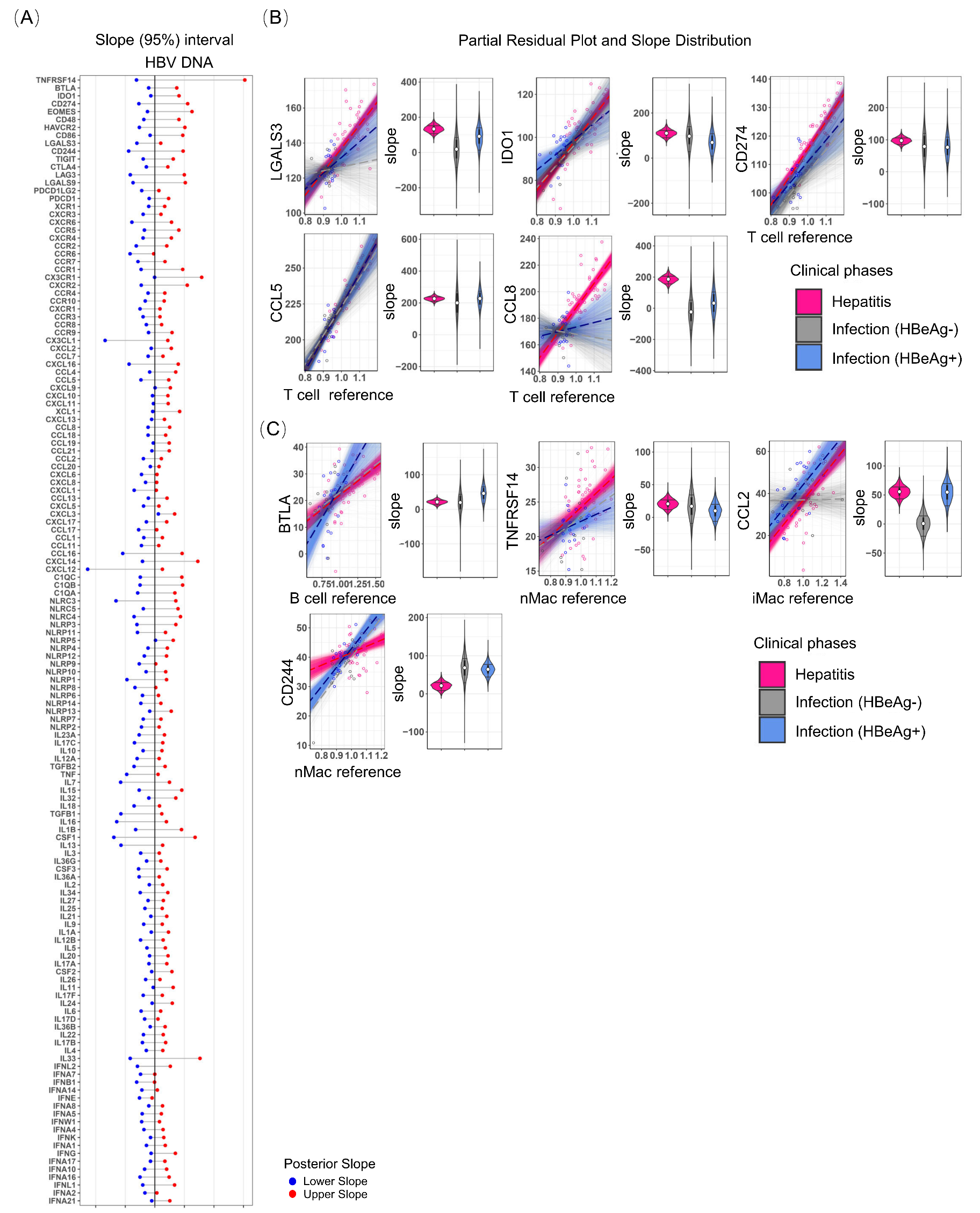


**Figure S6. Correlation between HBV DNA level and up-regulated genes’ expression in the phase of Hepatitis**

(A) 95% credible intervals of slopes of HBV DNA level with expression of genes from immune regulation, chemokines, complements, cytokines and interferons (rows). Colored bubbles indicate clearly positive slopes (red) or clearly negative slopes (blue). (B) Partial residual plot with fitted posterior lines and violin plot of the slope distributions from the fitted lines in chronic hepatitis (red), chronic infection (HBeAg+, blue), (HBeAg-, grey) for gene expression contribution by T cell. (C) Partial residual plot with fitted posterior lines and violin plot of the slope distributions from the fitted lines in chronic hepatitis (red), chronic infection (HBeAg+, blue), (HBeAg-, grey) for gene expression contribution by B cells, nMac, iMac cells.

**Figure S7**


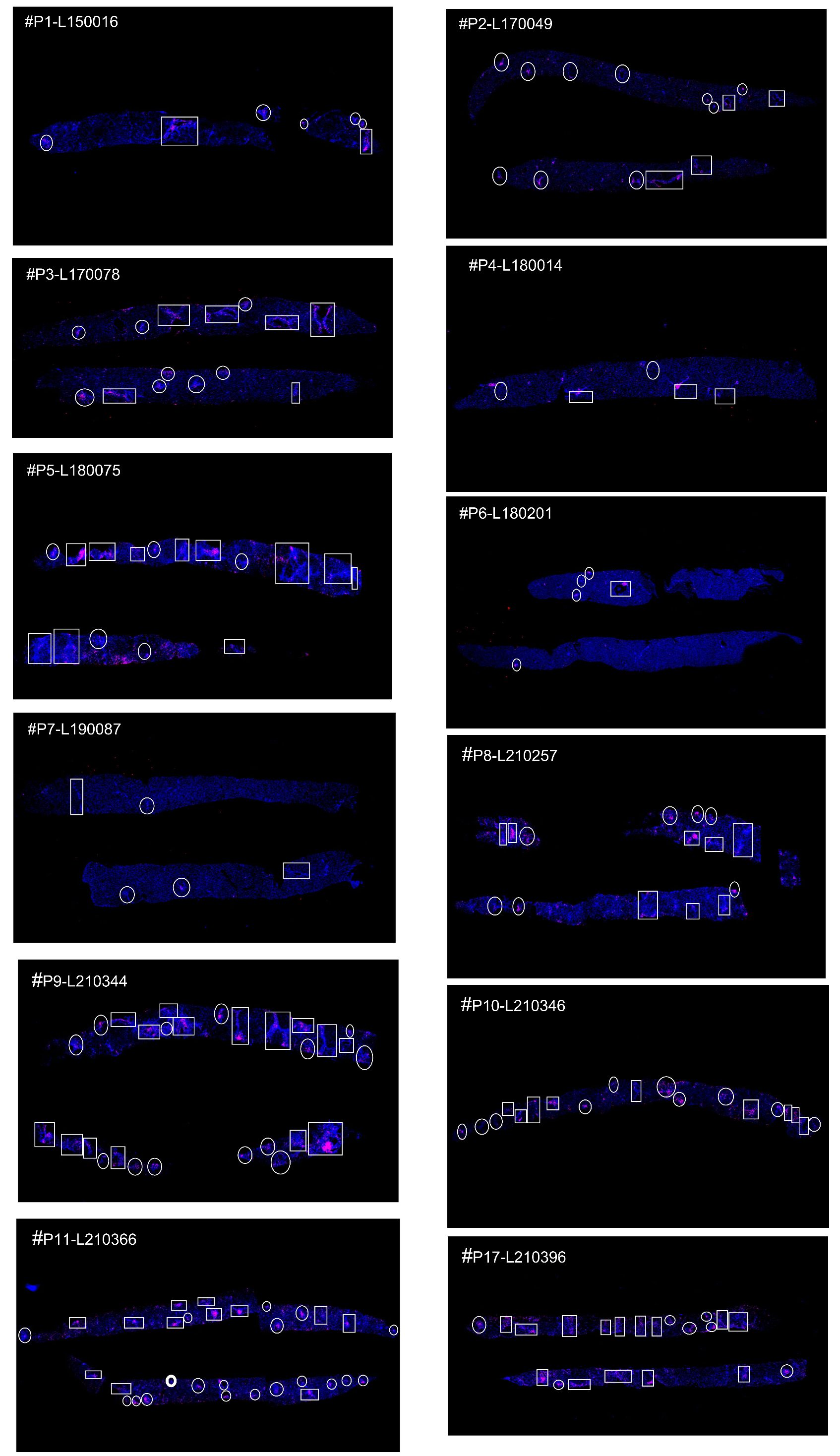


**Figure S7.** **Multiplex immune staining on liver slides and infiltrated area of HBeAg+ hepatitis patients**

Liver slides and infiltrated area of 12 HBeAg+ hepatitis patients.

**Figure S8**


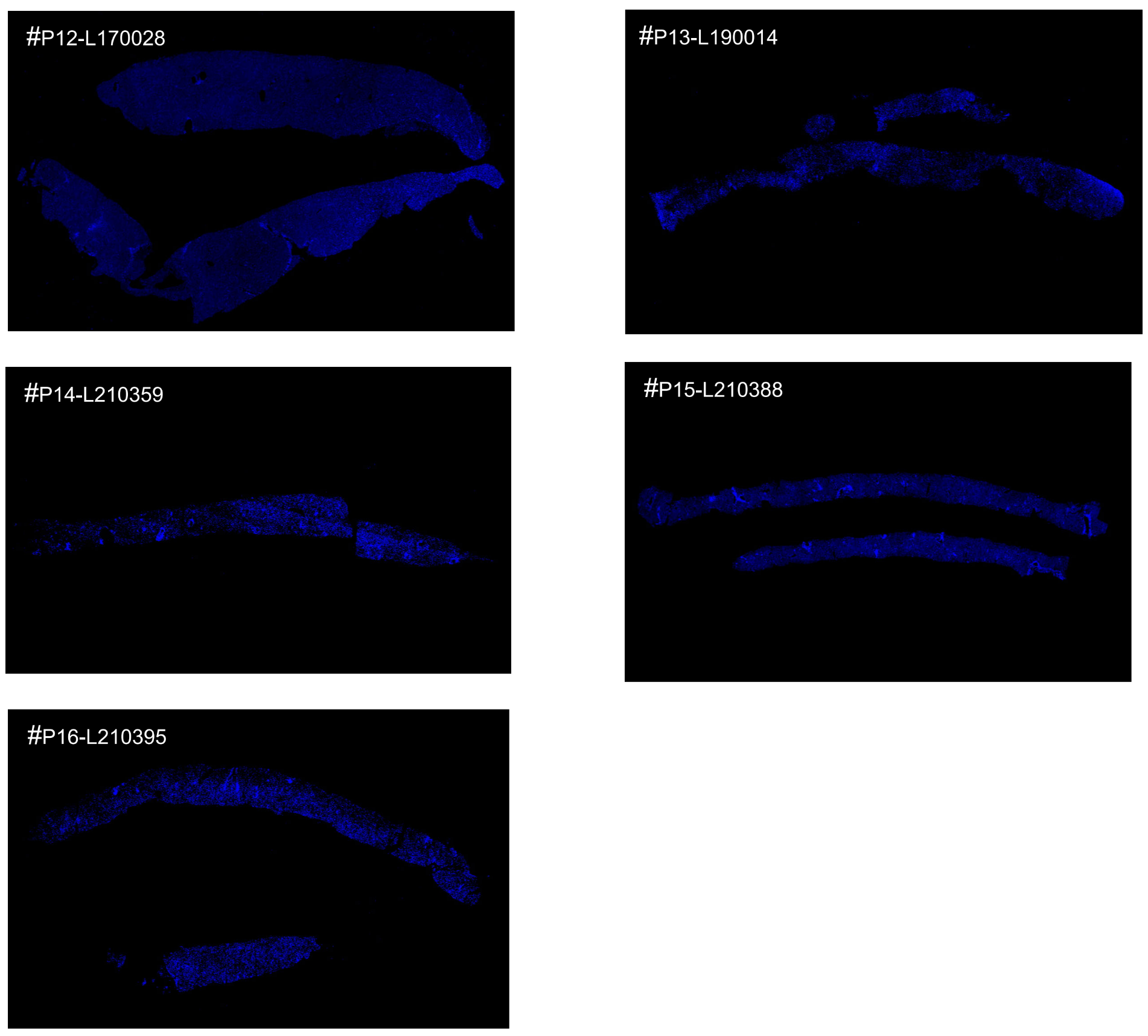


**Figure S8.** **Multiplex immune staining on liver slides of HBeAg+ infection patients**

Liver slides of 5 HBeAg+ infection patients.

**Figure S9**


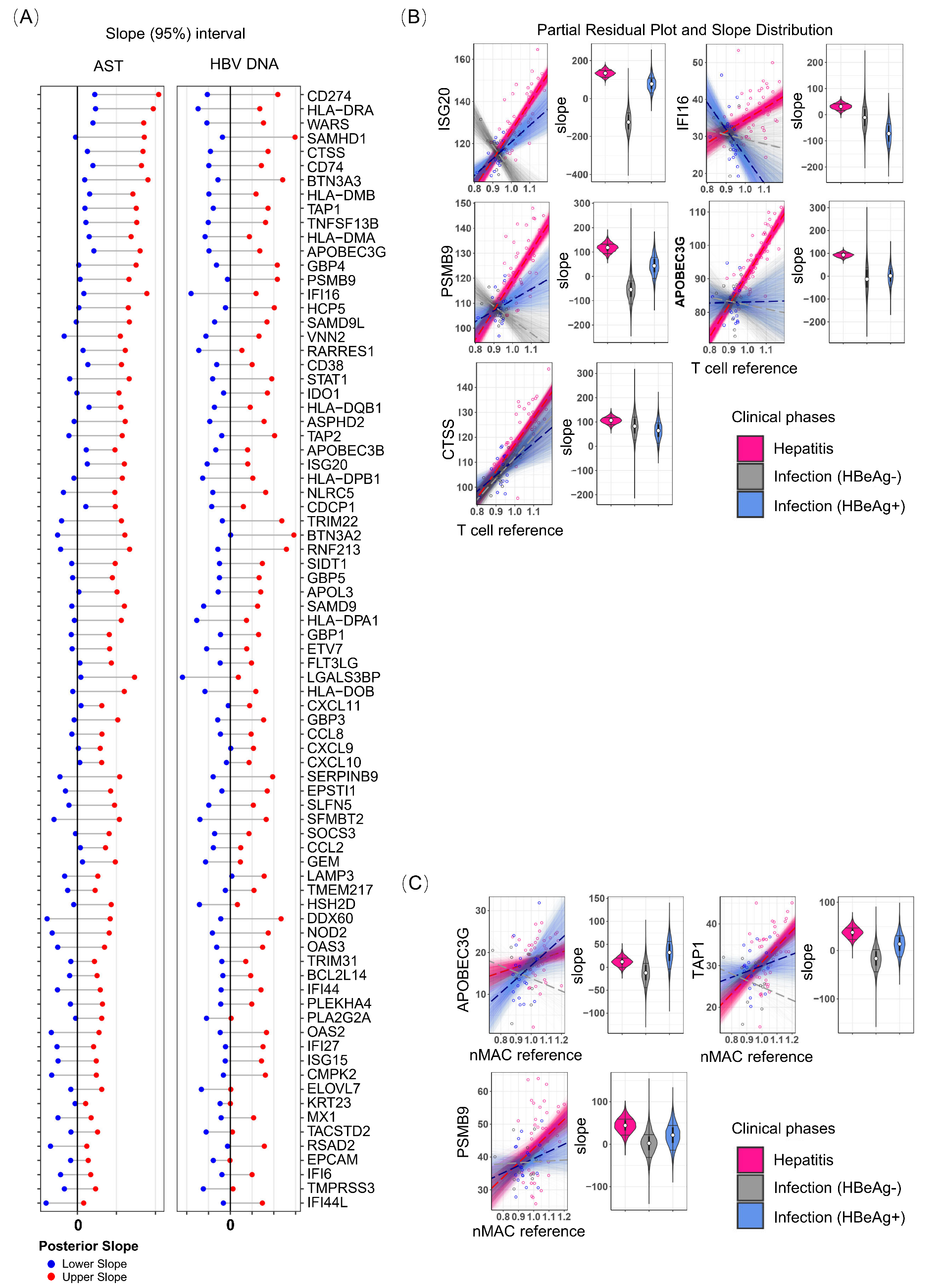


**Figure S9.** **Correlation between HBV DNA level, AST level, and up-regulated genes’ expression in the phase of Hepatitis**

(A) 95% credible intervals of slopes of AST (left panel) and HBV DNA (right panel) with expression of genes. Colored bubbles indicate clearly positive slopes (red) or clearly negative slopes (blue). (B) Partial residual plot with fitted posterior lines and violin plot of the slope distributions from the fitted lines in chronic hepatitis (red), HBeAg+ chronic infection (blue), HBeAg- chronic infection (grey) for gene expression contribution by T cell. (C) Partial residual plot with fitted posterior lines and violin plot of the slope distributions from the fitted lines in chronic hepatitis (red), HBeAg+ chronic infection (blue), HBeAg- chronic infection (grey) for gene expression contribution by nMac cells.
